# SARS-CoV-2 membrane protein recruits PP2A to dephosphorylate the nucleocapsid and promote virion production

**DOI:** 10.1186/s12929-026-01255-w

**Published:** 2026-05-15

**Authors:** Sheng-Han Wang, Tai-Ling Chao, Chi-Ling Hsieh, Pei-Jer Chen, Sui-Yuan Chang, Shiou-Hwei Yeh

**Affiliations:** 1 Hepatitis Research Center, National Taiwan University Hospital, Taipei, Taiwan; 2 Department of Clinical Laboratory Sciences and Medical Biotechnology, National Taiwan University College of Medicine, Taipei, Taiwan; 3 Graduate Institute of Clinical Medicine, National Taiwan University College of Medicine, Taipei, Taiwan; 4 NTU Centers of Genomic and Precision Medicine, National Taiwan University College of Medicine, Taipei, Taiwan; 5 Department of Internal Medicine, National Taiwan University Hospital, Taipei, Taiwan; 6 Department of Laboratory Medicine, National Taiwan University Hospital, Taipei, Taiwan; 7 Department and Graduate Institute of Microbiology, National Taiwan University College of Medicine, Taipei, Taiwan

**Keywords:** SARS-CoV-2, Nucleocapsid, Dephosphorylation, Virion secretion, Membrane protein, PP2A phosphatase, Coronavirus

## Abstract

**Background:**

The nucleocapsid (N) protein of coronavirus harbors a conserved serine/arginine (SR)–rich motif whose phosphorylation by GSK-3 is essential for viral transcription and replication. Our previous studies in Severe acute respiratory syndrome coronavirus 1 (SARS-CoV-1) and the JHM strain of mouse hepatitis virus revealed a phosphorylation-to-dephosphorylation transition during the viral life cycle, with newly synthesized N highly phosphorylated and virion-associated N hypophosphorylated. Here, we characterize this transition in SARS-CoV-2 and define its functional significance.

**Methods:**

Utilizing high-resolution gel analysis and the phospho-specific antibody, the phosphorylation levels of SARS-CoV-2 N proteins in the virus-infected Calu-3 cells and secreted virions from culture supernatants were compared between different viral strains. This phosphorylation-to-dephosphorylation transition was also verified in the SARS-CoV-2 virus-like particle (SC2-VLP) platform expressing N, membrane (M), envelope, and spike proteins. In this VLP system, the substantial contribution of N dephosphorylation status to particle secretion, either by blocking GSK-3 activity or phospho-related mutants, was assessed. With different viral structural protein-expressing clones and phosphatase inhibitors, we characterized the key viral factor and host phosphatase to mediate the N dephosphorylation process. Furthermore, we evaluated the antiviral efficacy of phosphatase inhibitors using the virus-infected Calu-3 culture.

**Results:**

Our results showed that the phosphorylation change of SR motif in N was observed across multiple strains and recapitulated in the SC2-VLP system. GSK-3 inhibition or a phospho-deficient N mutant, but not a phospho-mimetic one, enhanced VLP release, indicating that N dephosphorylation promotes virion secretion. Mechanistically, the M protein, via its C-terminal domain, interacts with phosphorylated N to recruit protein phosphatase 2A (PP2A) to the ERGIC, facilitating N dephosphorylation. Inhibition of PP2A, either by inhibitors or siRNA, impaired the M-induced N dephosphorylation, thereby suppressing viral assembly and progeny virion production. Blockade of PP2A also indirectly reduced N phosphorylation through the Akt-mediated inhibition of GSK-3, resulting in decreased genomic RNA synthesis.

**Conclusions:**

Collectively, these findings establish N dephosphorylation as a critical regulatory step in SARS-CoV-2 assembly and virion release. In ERGIC, this M protein-mediated process recruits host PP2A to dephosphorylate N, thus supporting PP2A as a promising pan-coronavirus antiviral target.

**Graphical Abstract:**

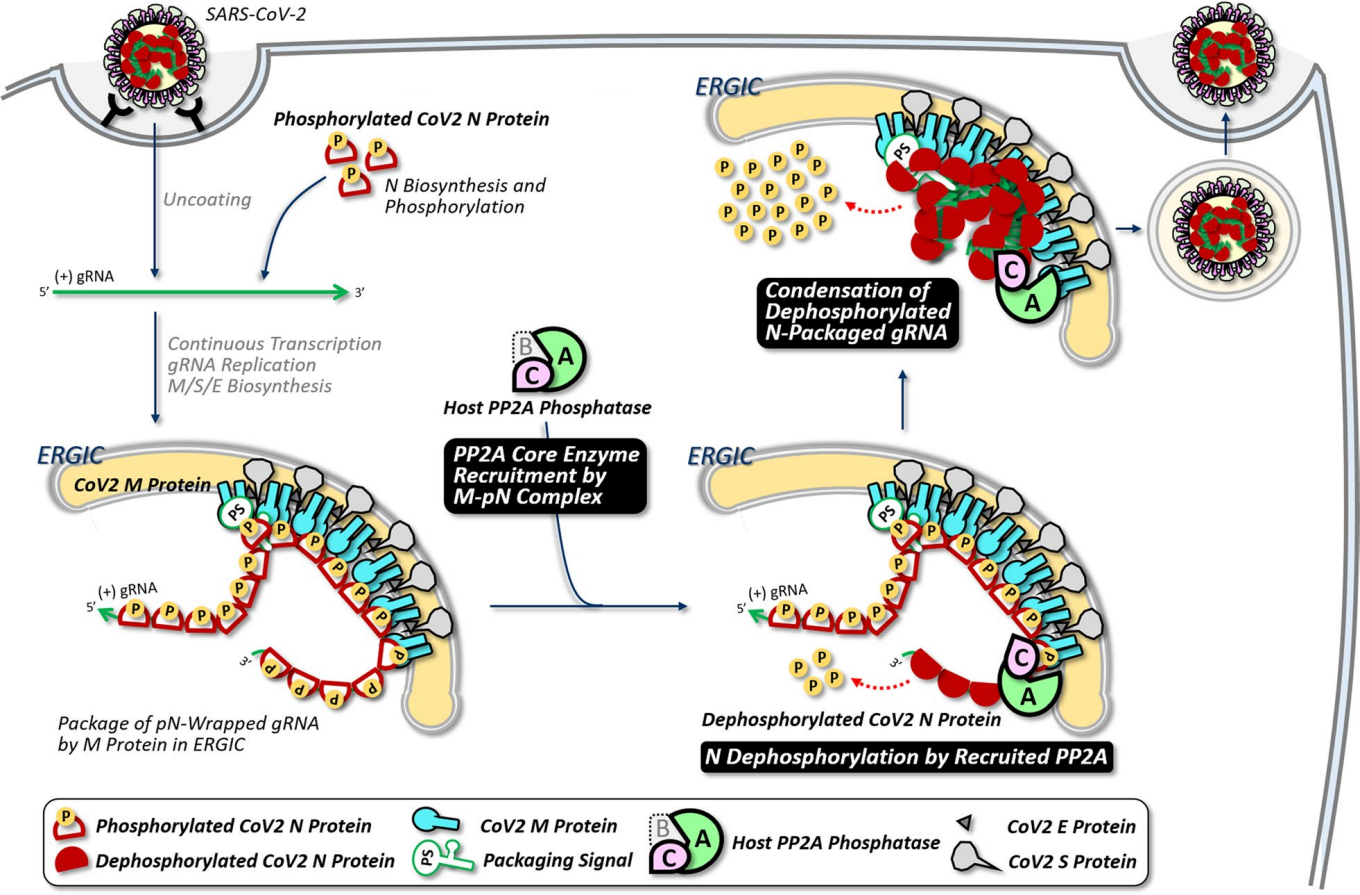

**Supplementary Information:**

The online version contains supplementary material available at 10.1186/s12929-026-01255-w.

## Introduction

Severe acute respiratory syndrome coronavirus 2 (SARS-CoV-2), the causative agent of the COVID-19 pandemic, is a highly transmissible lineage B member of the betacoronavirus (β-CoV) genus in the *Coronaviridae* family. It is a positive-sense, single-stranded RNA virus with a nonsegmented genome of approximately 30 kb [1]. This large genome is encapsulated within a crown-shaped virion, 80–90 nm in diameter, and encodes four major structural proteins: spike (S), envelope (E), membrane (M), and nucleocapsid (N) [2].

The viral N protein is evolutionarily conserved in CoVs and is one of the most abundant viral components in infected cells [3]. Structurally, the N protein contains two highly basic N-terminal and C-terminal domains with RNA-binding capacities. Between these two domains, there is a central linker region that is intrinsically disordered and contains serine/arginine (SR)-rich clusters [4]. Our previous studies demonstrated for the first time that the serine residue clusters within the SR-rich motif are the major phosphorylation sites for the N proteins of Severe acute respiratory syndrome coronavirus 1 (SARS-CoV-1) and the JHM strain of mouse hepatitis virus (JHMV), and that glycogen synthase kinase-3 (GSK-3) is one of the corresponding kinase responsible for phosphorylation [5, 6]. This regulatory mechanism has been further verified to be conserved in most CoVs, including Middle East respiratory syndrome coronavirus, SARS-CoV-2 and other CoVs [7, 8].

Phosphorylation of the N protein is crucial for recruiting the host helicase DDX1 to the viral ribonucleoprotein complex. In this context, N protein phosphorylation helps overcome template switching and reinforces the viral polymerase machinery to read through the body transcription regulatory sequences, enabling the biosynthesis of longer subgenomic RNAs (sgRNAs) and genomic RNAs (gRNAs) [5]. Recently, phosphorylation of the SARS-CoV-2 N protein has also been verified to be essential for viral replication [9]. Therefore, phosphorylation of the SR motifs in CoV N proteins is considered a conserved step required for their functions in viral replication.

Notably, our previous studies revealed distinct patterns of N phosphorylation status across different stages of viral life cycle. In SARS-CoV-1 and JHMV, the SR motifs of newly synthesized N proteins are highly phosphorylated in infected cells but become hypophosphorylated in secreted virions [5, 6]. This transition of N protein from the phosphorylated status to a dephosphorylated form may occur after viral genome replication, most likely during subsequent packaging and virion assembly, as reported in other viruses [10–12]. However, this hypothesis and its underlying regulatory mechanisms have not yet been investigated. Thus, in this study, we focus primarily on SARS-CoV-2 to address this issue.

The CoV M protein is a dimeric transmembrane protein localized in the ER-Golgi intermediate compartment (ERGIC), where it orchestrates virion assembly by interacting with N, E, and S proteins [13]. Its cytoplasmic C-terminal domain (CTD) is crucial for binding N and recognizing the genome packaging signal (PS) in full-length gRNA [14–16]. Deletion of the CTD abolishes virus-like particle (VLP) formation and recombinant virion production [17], highlighting its essential role in viral genome encapsidation and virion assembly.

A key step in this M-mediated morphogenesis involves the N protein, which normally exists in a relatively loose and dynamic state. When virion assembly is initiated, N undergoes a transition into a more condensed form. This condensation is thought to compress the ~ 30 kb wrapped gRNA, allowing it to be efficiently packaged through interaction with the M protein [18]. In vitro studies have recapitulated this process, showing that the CTD of the SARS-CoV-2 M protein drives phase separation of N, converting it from a liquid-like to a gel-like state [15]. Notably, the N mutants with phosphorylation-defective changes, such as serine-to-alanine substitutions within the SR motif or deletion of phosphorylation clusters, as well as treatment with GSK-3 inhibitors, also promote N condensation [15]. These findings suggest that dephosphorylation of N occurs during M-driven encapsidation, likely helping balance electrostatic charge for gRNA packaging and resulting in a hypo-phosphorylated state of N in secreted virions.

Since CoV M proteins lack functional phosphatase domains for N dephosphorylation [19], specific host phosphatases (PPases) are likely recruited to the viral encapsidosome to dephosphorylate N. Indeed, a recent interactome study in the SARS-CoV-2 infected cells revealed that the M protein associates with some PPases such as PP1, PP2A, and PP6 [20]. We thus hypothesized that the SARS-CoV-2 M protein facilitates the dephosphorylation of gRNA-associated N by recruiting specific PPase(s), promoting gRNA packaging and virion maturation in ERGIC. In this study, we validated the phosphorylation–dephosphorylation cascade of SARS-CoV-2 N protein and identified host PP2A as the M-recruited PPase critical for gRNA encapsidation and virion release, representing a potential new antiviral target.

## Materials and methods

### Plasmids and chemicals

The plasmid expressing the FLAG-tagged SARS-CoV-1 nucleocapsid protein was constructed in our previous study [6]. The full-length construct of SARS-CoV-2 nucleocapsid was established by cloning the cDNA fragment into the BamHI/NotI site of pcDNA3.1 vector. The insert was prepared and amplified by PCR, using the template which was reversely transcribed from the viral RNAs (Accession number: MN908947.3). To introduce specific mutations into the SARS-CoV-2 N protein-expressing clone, such as S188A, S206A, S188D or S206D, the QuikChange II XL Site-Directed Mutagenesis Kit (Agilent) was applied by using the following primer sets. N-S188A-F/R, N-S206A-F/R, N-S188D-F/R, and N-S206D-F/R (Supplementary Table 1). The constructs expressing SARS-CoV-2 M or E proteins (pLVX-EF1alpha-SARS-CoV-2-M or E) were provided by Dr. Che Ma (Academia Sinica, Taiwan). To establish the HA-tagged constructs expressing the full length (FL), the C-terminus truncated or internally deleted mutants of M protein, individual cDNA segments (M-FL, ∆195–222, ∆159–222, ∆119–222 and ∆119–158) were amplified by PCR and sub-cloned into the pcDNA-3xHA vector. The CoV2-M-IRES-E plasmid which expresses M and E proteins simultaneously was bought from Addgene (#177938). The plasmid encoding the humanized spike protein with D614G mutation was constructed from the HA-tagged spike clone as described previously [21], by introducing the stop codon to get rid of the C-terminal tag. For preparation of the virus-like particles containing the packable luciferase (Luc) transcript, the T20 construct which encodes the Luc mRNA fused with the packaging signal-containing segment (T20) of SARS-CoV-2 genome at the 3’-UTR was obtained from Addgene (#177941). For blockade of GSK-3 kinase activity, 6-bromoindirubin-30-oxime (BIO), LY2090314 (LY), and kenpaullone (Kenp) were purchased from MedChemExpress. The pan-PPase inhibitor okadaic acid and the PP2A-specific inhibitor fostriecin were obtained from Tocris Bioscience. Another pan-PPase inhibitor tautomycin and PP2A blocker LB-100 were ordered from MedChemExpress. All the compounds were prepared in DMSO solution for subsequent use, except for fostriecin and LB-100 which were dissolved in deionized water as stocks. All the detailed information of compounds used in this study are listed in Supplementary Table 2. The applied dosages of these compound as well as their reported cytotoxic ranges are summarized in Supplementary Table 3.

### Cell culture and transfection

Vero E6, DBT, Huh7 and 293T cell lines were all cultured in Dulbecco’s modified Eagle’s medium (DMEM, Invitrogen), supplemented with 10% heat-inactivated fetal bovine serum (FBS, Hyclone), glutamine (2 mM, Gibco) and penicillin/streptomycin/amphotericin B (100 unit/mL, 100 μg/mL, 0.25 μg/mL, respectively, Gibco), and incubated at 37 °C with 5% of CO2. The 293T-hACE2 cell line which stably expresses human ACE2 receptor was a gift obtained from Prof Mi-Hao Tao (Academia Sinica, Taiwan) and cultured as described above. Calu-3 cells were maintained in the 15% FBS-containing DMEM. For transfection of 293T cells, Lipofectamine 2000 (Thermo Fisher Scientific) was used to transfect plasmids into cells grown to 85–90% confluence by following the guidelines. Except for specific mention, the inhibitors were directly added into culture media at 4 h post-transfection with indicated concentrations, and cell lysates were harvested at 48 h post-transfection for subsequent analysis. All the detailed information and origins of cell lines used in this study are listed in Supplementary Table 2.

### Virus preparation and infection

The SARS-CoV-2 strains used in this study, including the D614G-related variants (NTU-03, 17 and 37), alpha variants (NTU-52, 54, 61 and 62), delta variants (NTU-96, 110, 119 and 121) and omicron variants [NTU-128 (B.1.1.529, BA.1), NTU-142 (BA.2.3.7, BA.2), NTU-293 (XBB.1.16)], were isolated from the sputum specimens of infected patients as described previously [22, 23]. The sequences of these isolates have been deposited in the GASAID with accession numbers, except for the strains of NTU-96, 119 and 121. For infection of SARS-CoV-2, Calu-3 cells were inoculated with the virus-containing media (MOI = 0.01) for 1 h. The cells were pretreated with inhibitors for 1 h prior to infection and continuously incubated thereafter to end point. At 24 h post-infection, the lysates and RNAs from infected cells and the virions in supernatants were harvested for subsequent analysis. For infection of JHMV into DBT cells (a well-established in vitro model for coronavirus research) and infection of HCoV-229E into Huh7 cells, the inoculation procedure and treatment timeline were followed as well as SARS-CoV-2. At 16.5 h post-infection, the culture media were collected for determination of viral loads. All the detailed information of viral strains used in this study are listed in Supplementary Table 2.

### Knockdown of phosphatase genes

The shRNA stocks were obtained from the National RNAi Core Facility (Academia Sinica, Taipei, Taiwan). The target sequences of these shRNA clones are listed as followed. shLuc, 5′-CAAATCACAGAATCGTCGTAT-3’; shPPP2C, 5′- TGGAACTTGACGATACTCTAA-3′; sh-PPP1C, 5′-TGAGTGCAAGAGACGCTACAA-3′; the clone 1 of shPPP2R1A, 5′-ACTGGATCCTGCTGCTGTAAT-3′, the clone 2 of shPPP2R1A, 5′- CTACGCTCTTCTGCATCAATG-3′; the clone 3 of shPPP2R1A, 5′-TTGCCAATGTCCGCTTCAATG-3′. The lentiviruses carrying these recombinant shRNAs were produced by co-transfecting 293T cells with pLKO.1-shRNA, the packaging vector pCMV-dR8.91, and the VSV-G expression vector pMD.G. At 48 and 72 h post-transfection, the virus-containing media were collected, followed by centrifugation and filtration (via 0.22 μm-filters) to remove cell debris. To knockdown cellular phosphatase gene expression, 293T cells were incubated with the lentivirus-containing media for 16 h, and then proceeded for transfection and molecular assay, with the shRNA targeting luciferase (shLuc) served as negative controls.

### Preparation of virus-like particles and subsequent assay

The SARS-CoV-2 empty VLPs were prepared by following the previously reported protocols with modification [24]. In brief, the plasmids expressing the SARS-CoV-2 N, M, E or humanized S protein were cotransfected at a molecular ratio of 1:1:1:1 into 293T cells via Lipofectamine 2000. 4 h later, the transfection reagents were washed out by PBS and fresh media were applied to incubate the transfected cells. The culture media collected at 48 h post-transfection were centrifuged (1,710 × *g*, 4°C, 10 min) and then filtered through a 0.22-μm filter to remove cell debris. The 9 mL of cleared media were finally transferred to a centrifuge tube with 1 mL of 20% sucrose (prepared in PBS) carefully loaded at the bottom for VLP cushion. After ultracentrifugation (106,910 × *g*, 4 °C, 20 h), the precipitates at the bottom of the tubes were resuspended in NET buffer (50 mM Tris, pH 7.5, 150 mM NaCl, 1 mM EDTA, 0.1% NP-40) for subsequent protein assay. On the other hand, to prepare the RNA-containing VLPs, the plasmid encoding the packable T20-Luc reporter RNA, together with the constructs expressing the N, M and E (both from the clone of CoV-2-M-IRES-E) and humanized S protein, were cotransfected at a molecular ratio of 3:1:1:1 into 293T cells (which were seeded at a density of 0.5 × 10^6 in 6 cm plates the day before transfection). After transfection for 4 h, the cells were washed by PBS and incubated with fresh media. At the period of 24 h to 48 h post-transfection, the culture media were collected for preparation of the Luc reporter RNA-containing VLPs by following the cushion process as well as empty VLPs. The obtained precipitates were then resuspended in 100 μL of fresh media for VLP infection assay. To evaluate the infectious capacities of these RNA-containing VLPs, the 293T-hACE2 cells (at a density of 0.1 × 10^6 in 24-wells) were inoculated with the equal volumes (30 μL) of VLP-containing media and then lysed at 24 h post-infection by passive lysis buffer (Promega). The concentrations of obtained cell lysates were determined by BCA Protein Assay Kit (Thermo Fisher Scientific) and the equal amounts of these lysates were used to examine the luciferase activity by Luciferase Reporter Assay System (Promega).

### Subcellular fractionation and sucrose gradient

To obtain fractionated cytosolic and membrane lysates, the transfected 293T cells were washed with cold PBS and processed using a subcellular protein fractionation kit (Thermo Fisher Scientific) according to the guidelines. For isolation of ERGIC-enriched compartments from sequential centrifugation at different speed, the modified procedure based on the previous report was applied [25]. Briefly, the cell pellets collected at 48 h post-transfection were resuspended in the hypotonic buffer (0.25 M Sucrose, 20 mM HEPES, pH 7.4, 1 mM EDTA) and proceeded to freeze and thaw for 5 cycles. Breakdown of cell membrane was confirmed by Trypan Blue staining. The homogenates were subjected to sequential centrifugation, from 1,000 × *g* (10 min), 3,000 × *g* (10 min), to 20,600 × *g* (30 min). The final sedimented compartments (20,600 × *g*) were lysed by RIPA lysis buffer (Merck Millipore), while the final supernatants were proceeded through centrifugal concentrator (10K MWCO, Merck) to obtain the concentrated sample. The lysates derived from the last round of sedimentation (20,600 × *g*) were served as the ERGIC-enriched parts, as well as the concentrated supernatants (cytosol parts), were used for subsequent protein analysis. To examine the subcellular localization of SARS-CoV-2 N/M proteins and host PP2A by sucrose gradient, the analysis was conducted by ultracentrifuge, using the modified procedure based on our previous study [10]. Briefly, the transfected 293T cells (at 48 h post-transfection) were homogenized in isotonic buffer (0.25 M sucrose, 10 mM HEPES, pH 7.4, and 10 mM EDTA) by passing through a syringe (26G) quickly and repeatedly for four times. After removing cell debris by centrifugation (500 × *g*, 4 °C, 10 min), the supernatants were overlaid onto a sucrose gradient, which was constituted by laying 50, 40, 30, 20 and 10% sucrose layers (prepared in 10 mM HEPES, pH 7.4, 10 mM EDTA) from bottom to top. After ultracentrifugation (230,000 × *g*, 16°C, 22 h), 13 fractions were collected and equal volumes of these samples were used for subsequent immunoblot assay. To analyze the released pseudo-particles, the PBS-resuspended VLPs prepared from the cushioned media of producing 293T cells were layered on the top of sucrose gradient (10–50%, prepared in PBS). Following ultracentrifuge, equal volumes of 13 fractionated samples were analyzed as described above.

### Plaque assay

To determine the viral titers of SARS-CoV-2 in the supernatants of infected cells, a plaque assay was performed as previously described [21]. In brief, the cell monolayers of Vero E6 in 24-well plates were incubated with serially diluted viruses for 1 h at 37 °C. After removal of the inoculum, the infected cells were maintained in DMEM supplemented with 1% methylcellulose for 5 days, after which the cells were fixed with 10% formaldehyde and stained with 0.5% crystal violet. The viral titers were determined by counting plaques and calculating the means from three independent experiments. The results were presented as averages with standard deviations and shown as the log copies per mL, along with the vehicle control set as 100% for relative percentage comparison. Significant differences between paired groups were examined by Student’s t test.

### Quantitative reverse transcription polymerase chain reaction and northern blotting

To measure the viral loads of SARS-CoV-2 or T20-Luc RNA-containing VLPs in the culture media, the soups collected in indicated time periods were first treated with micrococcal nuclease (New England Biolabs) to remove any particle-free nucleic acids by incubation at 37 °C for 30 min. Then, the packaged RNAs of secreted virions or VLPs in culture supernatants were extracted by NucleoSpin RNA extraction kit (Macherey-Nagel). The obtained RNAs were reversely transcribed (RT) into cDNAs, using the SuperScript III First-Strand Synthesis System (Thermo Fisher Scientific). The levels of SARS-CoV-2 gRNAs from secreted virions or T20-Luc RNAs from VLPs were determined by quantitative polymerase chain reaction (qPCR), using the primer sets, E-Sarbeco-F/R [26] or 20914-F/21223-R, respectively. To determine the viral loads of JHMV and HCoV-229E in culture media, the viral genomic RNAs were extracted from secreted virions and reversed transcribed into cDNAs by following the RT-qPCR procedure as described above. The primer sets, JHMV-F/R and 229E-1ab-F/R, were used to determine the titers of JHMV and HCoV-229E, respectively. For quantification of viral gRNA levels in host cells, the total RNAs extracted from infected Calu-3 cells were processed as described above for RT-qPCR analysis, using the primer set (20914-F/21223-R) targeting ORF-1ab. Quantification of PBGD mRNA levels (by PBGD-1070F/1266R) serves as internal controls. For Northern blotting, it was conducted as described in our previous study [21], with the HindIII-digested lambda phage DNA (New England Biolabs) as an electrophoretic marker. In brief, 1 μg of the extracted total RNAs from infected Calu-3 cells were denatured (by 2% Formaldehyde and heated at 65 °C for 10 min) and separated in an 1% formaldehyde-agarose gel (by 70 Volt for 5 h). After electrophoresis, the gel was soaked in NaOH (50 mM) to breakdown large RNA molecules, and then neutralized by Tris–HCl (pH 7.5, 100 mM) for 30 min, followed by incubation in 20 × SSC buffer (3M NaCl, 0.3M sodium citrate, pH 7.0) for another 20 min. Finally, by capillary transfer via salt bridge, the RNAs in the processed gel was transferred to a nylon membrane with positive charge (Hybond-N, Amersham Biosciences) for overnight. Finally, the obtained blot was UV-crosslinked (1800 × 100 μJ/cm^2^) and hybridized with the digoxigenin (DIG)-labeled SARS-CoV-2 N probe (50 °C, overnight), which detected viral gRNAs and sgRNAs simultaneously by recognizing the viral N gene. After washing and blocking the northern blot by DIG Wash and Block Buffer set (Roche Diagnostics), the signals were output upon the application of Anti-Digoxigenin-AP Fab Fragments (Roche Diagnostics) and luminescent CDP-Star (Roche Diagnostics) stubstrates. The mRNA amounts of cellular porphobilinogen deaminase (PBGD) genes in each samples served as loading controls by using the corresponding probes for detection. To prepare the DIG-labelled probes for recognition of SARS-CoV-2 N and PBGD genes, the primer sets of CoV-N-1F/R and PBGD-F/R, which had been reported in our previous work [21], were utilized to produce the materials by PCR using the PCR DIG probe synthesis kit (Roche Diagnostics). The info of primer sets used for RT-qPCR or probe synthesis are summarized in Supplementary Table 1. All the detailed information of experimental reagents are listed in Supplementary Table 2.

### Cell lysate preparation and western blotting

At 48 h post-transfection, the Vero E6 or 293T cells were lysed by NET buffer plus Protease Inhibitor Cocktail (Roche Diagnostics) and Phosphatase Inhibitor Cocktail II (Merck Millpore) to obtain crude cell lysates. For the SARS-CoV-2-infected Calu-3 cells, RIPA lysis buffer (Merck Millipore) was applied to completely lyse the cells and denature proteins. The concentration of debris-removed cell lysate (by centrifugation, 15,490 × *g*, 4 °C, 10 min) was determined by BCA Protein Assay Kit (Thermo Scientific). For in vitro dephosphorylation, 20 μg of the NET lysates were treated with 10 unit of alkaline phosphatase (Thermo Fisher Scientific) by incubation at 37 °C for 2 h. After treatment, the lysates were proceeded for electrophoresis in 10% Bis-Tris NuPAGE (1.0-mm, Thermo Fisher Scientific) high resolution gel and then analyzed by standard immunoblotting. Blotting data shown are represenative of 2–3 independent experiments. The dencitometry of protein signals were determined by ImageJ to evaluate their relative amounts. All the detailed information of experimental reagents and primary antibodies are listed in Supplementary Table 2.

## Coimmunoprecipitation assay

Coimmunoprecipitation (CoIP) was performed to assess the interaction between viral N/M proteins and catalytic subunit of PP2A. Briefly, the 293T cells co-transfected with the plasmids encoding N and/or M protein were lysed by NET buffer at 48 h post-transfection. 500 μg of cleared cell lysates were incubated with anti-N antibodies for overnight at 4 °C with gentle rotation. Immune complexes were then captured by incubation with Protein G agarose beads for an additional 1 h at 4 °C. The beads were washed for 3 times with NET buffer, and then bound complexes were eluted by boiling in SDS sample buffer, followed by electrophoresis and immunoblotting for protein detection.

## Results

### SARS-CoV-2 N protein is hypophosphorylated in secreted virion

**Fig. 1:**
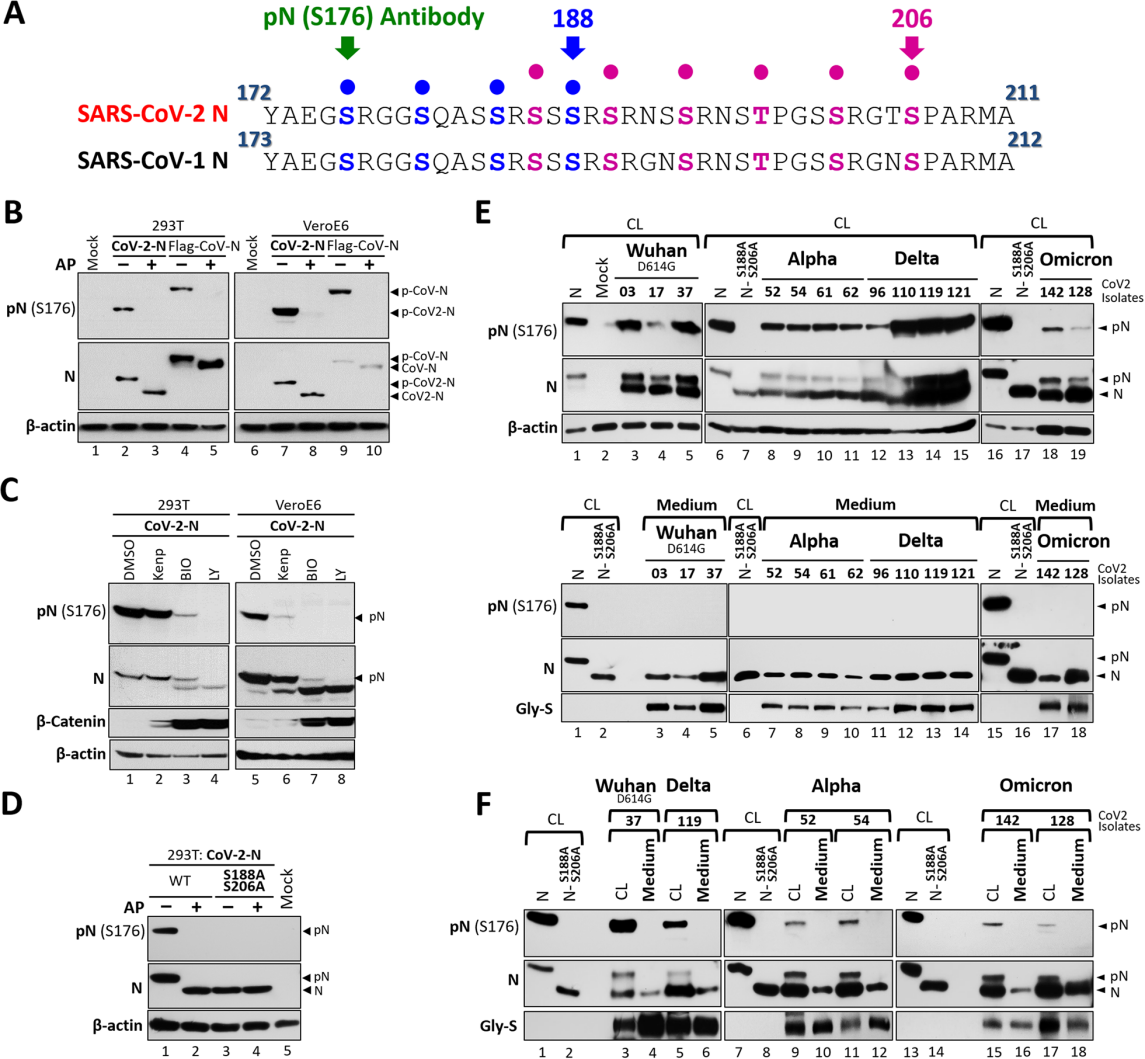
The SARS-CoV-2 N Protein Is Hypophosphorylated in Secreted Virions. **A** Comparison of phosphorylation stretches in the N proteins of SARS-CoV-1 and SARS-CoV-2. The antibody recognition residue (previously referred to as pN-S177 Ab, but renamed here as pN-S176 Ab) is also indicated. **B** Lysates from 293T or Vero E6 cells transfected with constructs expressing the N proteins of SARS-CoV-2 (CoV-2-N) or SARS-CoV-1 (Flag-CoV-N) were incubated with alkaline phosphatase (AP), followed by high-resolution gel electrophoresis and western blot analysis of phosphorylated and dephosphorylated N proteins **C, D** As described in (**B**), lysates derived from cells treated with the GSK-3 inhibitors (Kenp, Kenpaullone; Bio, 6-bromoindirubin-30-oxime; and LY, LY2090314) (**C**), or from cells expressing the phospho-deficient mutant (N-S188A/S206A) followed by AP treatment (**D**), were analyzed to assess the phosphorylation status of the N protein. **E** Cell lysates (CL) and culture media collected from Calu-3 cells infected with different SARS-CoV-2 variants (MOI = 0.01) at 24 hrs post-infection were analyzed as described in (**B**). Lysates from 293T cells overexpressing the wild-type N protein or the S188A/S206A mutant served as the phosphorylated and unphosphorylated controls, respectively. **F** Similar to (**E**), cell lysates and culture media prepared from Calu-3 cells infected with different SARS-CoV-2 strains were examined side by side. pN, phosphorylated N proteins. Gly-S, the glycosylated form of the spike protein. Data are representative of three independent experiments

Distinguishing the phosphorylated status of SARS-CoV-2 N protein from its dephosphorylated status is essential for monitoring its replication-stage modifications. The SR-rich motif (residues 172–211) of SARS-CoV-2 N shows highly conservation with SARS-CoV-1 (37/40 residues) (Fig. 1A), indicating that the GSK-3-mediated phosphorylation mechanism identified previously [6] also applies to SARS-CoV-2, with priming sites at S188 and S206. Using our established protocol [6], we have tried to resolve phosphorylated and dephosphorylated SARS-CoV2-N proteins by high-resolution NuPAGE and detected them with a phosphorylation-specific antibody.

High-resolution NuPAGE analysis of the lysates from transfected 293T and Vero E6 cells separated the phosphorylated and dephosphorylated SARS-CoV-2 N proteins as well as those of SARS-CoV-1 (Fig. 1B–D, middle panels). Alkaline phosphatase pretreatment yielded a faster-migrating band, showing that the N proteins are predominantly phosphorylated (Fig. 1B, lanes 3 and 8 vs. lanes 2 and 7). Similar mobility shifts were also observed with GSK-3 inhibitor treatment (Kenpaullone, BIO, LY2090314) in a dose-dependent manner, with efficacy indicated by accumulation of β-catenin, a GSK-3 substrate (Fig. 1C, middle panels). A comparable shift was also observed with the phosphorylation priming-defective mutant N-S188A/S206A (Fig. 1D, middle panel), supporting GSK-3 as a key kinase for N phosphorylation. We then tested the phosphorylated N-specific antibody (pN-S176 Ab) developed in our previous SARS-CoV-1 study [6], which cross-reacts with SARS-CoV-2 N at S176 (Fig. 1A). The antibody specifically recognized the upper bands in NuPAGE, which could be abolished by alkaline phosphatase treatment, GSK-3 inhibition, or mutation of the priming sites (N-S188A/S206A), thus validating its specificity for phosphorylated SARS-CoV-2 N protein (Fig. 1B–D, upper panels).

High-resolution NuPAGE analysis and the pN-S176 antibody were then used to assess the phosphorylation status of SARS-CoV-2 N proteins in cell lysates and secreted virions from the virus-infected Calu-3 cells, including the Wuhan strain (D614G), as well as the Alpha, Delta, and Omicron variants [1]. As controls, the lysates from 293T cells overexpressing either wild-type N (phosphorylated) or the S188A/S206A mutant (non-phosphorylated) were included. In infected cells, both phosphorylated and dephosphorylated forms of N proteins were detected (Fig. 1E, upper panel), whereas only the dephosphorylated form was found in culture media containing secreted virions, regardless of viral strains (Fig. 1E, lower panel). This observation was confirmed by side-by-side analysis of cell lysates and media (Fig. 1F). Thus, similar to other CoVs (SARS-CoV-1 and JHMV) reported in our previous studies [5, 6], the SARS-CoV-2 N protein also transits from an intracellular phosphorylated form to a hypophosphorylated state within extracellular virions.

## Dephosphorylated N enhances the release of virus-like particles

**Fig. 2:**
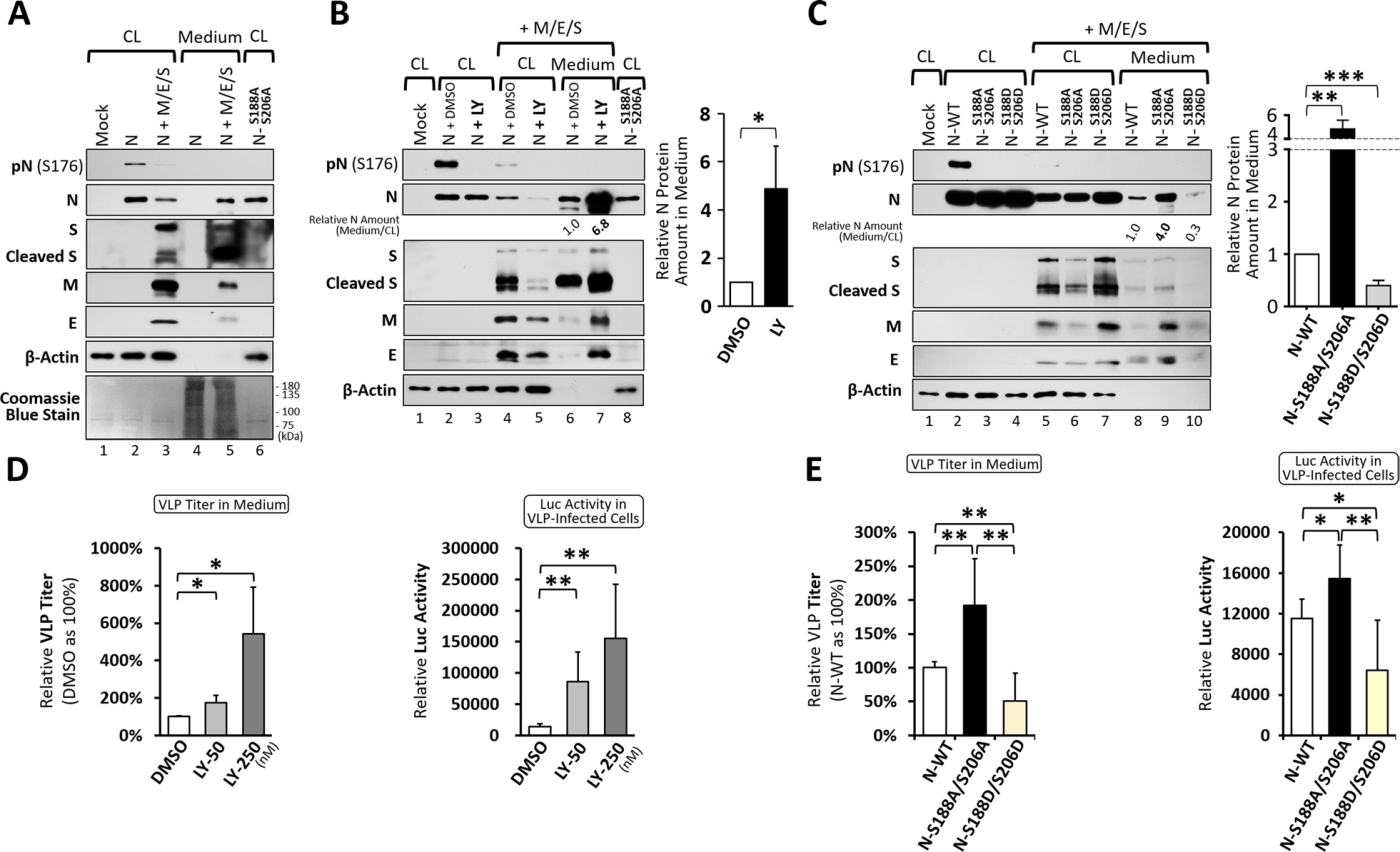
Dephosphorylation of the N Protein Enhances the Release of Virus-Like Particles.** A** Cell lysates (CL) and released VLPs in culture medium precipitates prepared from 293T cells expressing the indicated viral proteins were analyzed by standard SDS‒PAGE to evaluate SARS-CoV-2 N protein phosphorylation in the absence or presence of other structural proteins. **B**, **C** As described in (**A**), 293T cells were either treated with the GSK-3 inhibitor LY2090314 (LY, 250 nM) (**B**) or transfected to express phospho-related N mutants in combination (**C**), and then lysed at 72 h post-transfection for western blott analysis. The corresponding culture medium precipitates were evaluated in parallel (left panels). The relative amount of N protein in the medium compared to that in cell lysates (with the DMSO-treated control or wild-type N set to 1.0) was quantified from three independent experiments and is presented as mean ± SD (right bar graphs). **D**, **E** The titers of RNA (T20-Luc)-containing VLPs in culture supernatants collected from producing 293T cells, either treated with LY (50 or 250 nM) (**D**) or co-transfected with wild-type N or phospho-related mutants (**E**), were determined by RT-qPCR at 48 h post-transfection (left bar graphs). The infectivity of these VLPs (using equal volumes of medium) was assessed in 293T-hACE2 cells by a luciferase reporter assay at 24 h post-infection (right bar graphs). Relative RNA titers in culture medium and luciferase activities in infected cells were quantified from four independent experiments and are presented as mean ± SD. Blotting data are representative of three independent experiments. *p < 0.05; **p < 0.01; ***p < 0.001

To assess phosphorylation changes in the N protein during the late stage of viral assembly and maturation, and to evaluate their functional relevance, we used a surrogate virus-like particle (VLP) system [24]. The plasmids expressing structural proteins (N, M, E and S) were co-transfected into 293T cells to examine N abundance and phosphorylation status in both cells and secreted VLPs. When expressed alone, the N protein was highly phosphorylated and detected exclusively within cells, with no detectable levels in the culture media (Fig. 2A, lanes 2 and 4). However, upon co-expression with the other three structural proteins, N protein phosphorylation was dramatically reduced, accompanied by a moderate decrease in intracellular protein amount (Fig. 2A, lane 3 vs. lane 2). Meanwhile, the N protein could be detected in culture media along with other viral structural proteins (Fig. 2A, lane 5 vs. lane 4), most likely originating from secreted VLPs as previously reported [24, 27]. Most importantly, similar to the observations in authentic infection systems, the N protein derived from secreted VLPs was hypophosphorylated, showing no reactivity with the pN-S176 antibody (Fig. 2A, lane 5 vs. lane 2). These results demonstrated that this simplified VLP system, expressing only the N, M, E, and S proteins, faithfully recapitulated the phosphorylation-to-dephosphorylation transition of N protein following virion release, as observed in infected cells.

Next, to examine the effect of N dephosphorylation on virion secretion, the VLP-producing cells were treated with the GSK-3 inhibitor LY2090314 (LY), which nearly completely blocked intracellular N phosphorylation (Fig. 2B, lanes 3 and 5 vs. lanes 2 and 4). LY treatment increased extracellular N abundance in the culture media by 5-7 fold compared to vehicle controls, accompanied by elevated levels of other structural proteins, while their intracellular amounts decreased concordantly (Fig. 2B, lane 7 vs. lane 6, and lane 5 vs. lane 4). In a similar way, substituting wild-type N with the phospho-deficient mutant (N-S188A/S206A) enhanced the secretion of N protein, whereas the phospho-mimetic one (N-S188D/S206D) reduced it, both accompanied by parallel amount changes in other structural proteins (Fig. 2C). Notably, the intracellular N protein level of the N-S188A/S206A mutant did not decrease as observed with LY2090314 treatment, likely reflecting the broader effects of pharmacological kinase inhibition compared with site-specific mutation. Nevertheless, both approaches indicate that reduced N phosphorylation enhances N secretion in the presence of M/E/S proteins.

To further examine whether N dephosphorylation promotes the release of RNA-containing VLPs, the 293T cells were co-transfected with a construct encoding the luciferase RNA which contains the PS of SARS-CoV-2 (T20-Luc) [24], along with the plasmids expressing structural proteins as described above. LY treatment increased the release of RNA-containing VLPs by 2-5 fold in a dose-dependent manner (Fig. 2D, left graph). This increase was reflected by the higher luciferase activity in the VLP-infected cells correspondingly (Fig. 2D, right graph), confirming the enhanced secretion of RNA-containing VLPs into culture media. Similarly, the phospho-deficient N mutant (N-S188A/S206A) increased the release of RNA-VLPs and subsequent luciferase activity, whereas the phospho-mimetic mutant (N-S188D/S206D) reduced both (Fig. 2E). Transmission electron microscopy analysis showed that VLPs generated with WT or mutant N proteins were broadly similar in morphology, although phospho-deficient N (S188A/S206A) particles were slightly larger with less stain penetration, whereas phospho-mimetic N (S188D/S206D) particles were more heterogeneous and irregular, possibly reflecting differences in RNA content or particle integrity (Supplementary Fig. 1). Together, these results indicate that N dephosphorylation enhances the release of RNA-containing VLPs.

## The M protein mediates the dephosphorylation of N protein

**Fig. 3:**
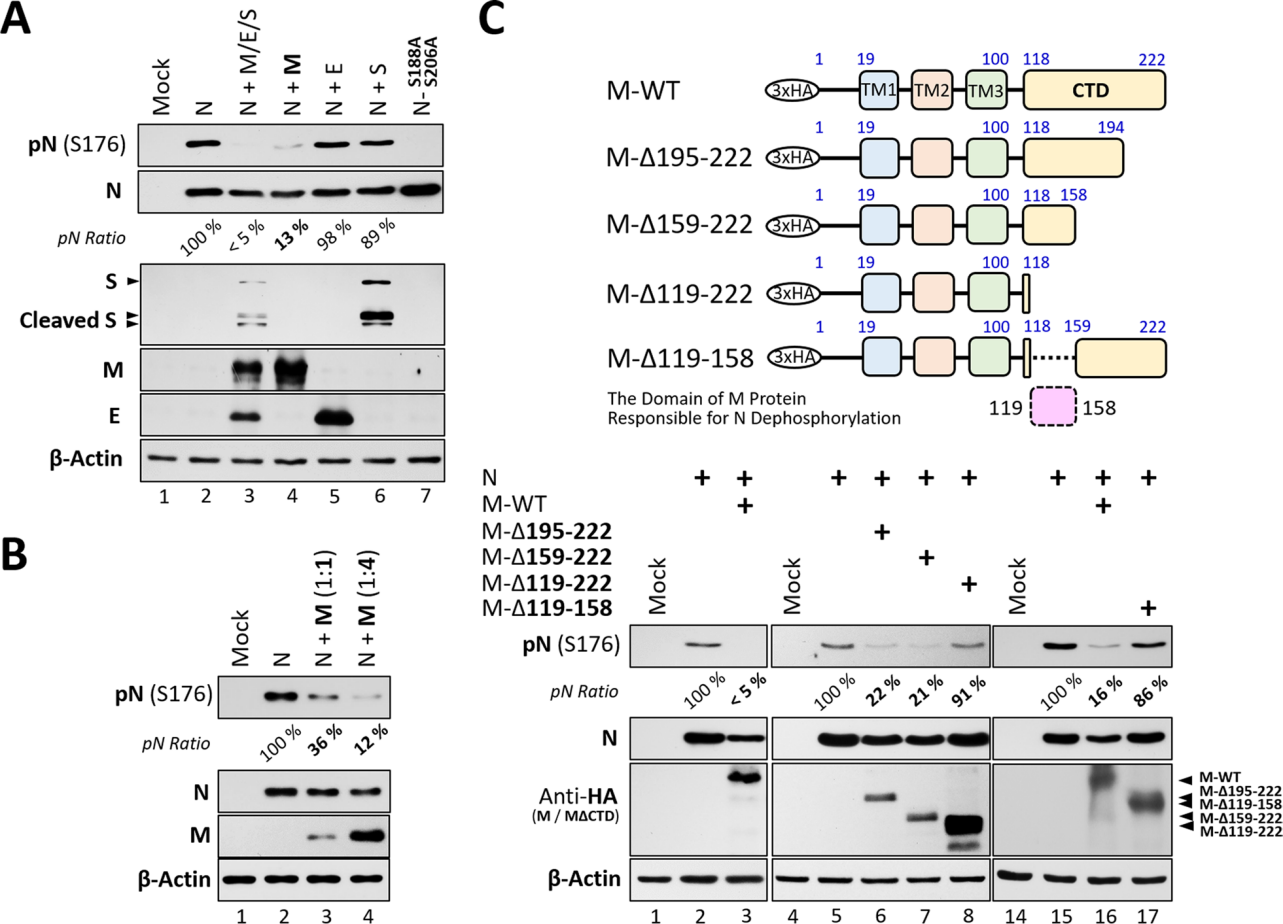
The M Protein Mediates the Dephosphorylation of the N Protein. **A** The phosphorylation status of the SARS-CoV-2 N protein expressed in 293T cells co-transfected with other viral protein constructs was determined by western blotting. The S188A/S206A N mutant served as the unphosphorylated control. **B** The phosphorylation level of the N protein in 293T cells co-expressing the M protein at different amounts (molar ratios of 1:1 or 1:4) was examined by western blotting. **C** Schematic illustration of HA-tagged constructs expressing M proteins with serial C-terminal truncations. The numbers above the diagrams indicate amino acid residues of the M protein (upper diagram). As in (**B**), the phosphorylation level of N was determined in 293T cells co-transfected with different M-ΔCTD constructs (with adjusted N/M molar ratios of 1:1, 1:2, 1:2, 1:0.5, and 1:0.5 for N plus WT-M, Δ195–222, Δ159–222, Δ119–222, and Δ119–158, respectively) by western blotting (lower panels). The pN ratios (phosphorylated N normalized to total N protein) were compared, with N expressed alone set to 100%. Data are representative of three independent experiments. TM, transmembrane motif; CTD, C-terminal domain

To identify which viral structural proteins mediate N dephosphorylation, the M, E, or S encoding clone was individually co-transfected with the N-expressing construct into 293T cells. The M protein alone, but not E or S, promoted N dephosphorylation as efficiently as co-expression of all three components (Fig. 3A, lanes 4–6 vs. lane 3). Notably, the M-induced N dephosphorylation was a dose-dependent manner relied on M protein amount (Fig. 3B, lanes 3–4 vs. lane 2).

The M protein is an integral transmembrane protein consisting of two functional domains connected by a hinge region. Its three N-terminal helices form a bundle-like structure that spans the lipid bilayer, anchoring the M protein at ERGIC membrane. The CTD of M protein comprises eight β-sheet motifs that fold into an inward-facing platform, enabling its interaction with the gRNA/N protein complex for genome packaging [13]. Deletion of the CTD completely disrupts the binding capacity of M to N protein [13].

To identify the M protein domain responsible for N dephosphorylation, we generated a series of C-terminus truncated mutants (Fig. 3C, top). Compared with full-length M (Fig. 3C, lane 3 vs. lane 2), deletion of the entire CTD (residues 119–222) abolished the M-dependent N dephosphorylation (Fig. 3C, lane 8 vs. lane 5), whereas partial deletions beyond residues 195 or 159 (Δ195–222 or Δ159–222) had little effect (Fig. 3C, lanes 6 and 7 vs. lane 5). Immunofluorescence (IF) staining showed that N is diffusely distributed in the cytosol when expressed alone. Upon co-expression with M, which predominantly localizes to the ERGIC (Supplementary Fig. 2A), partial colocalization of N with M-positive compartments was observed in approximately 80% of cells. In contrast, the CTD-truncated M mutant (M-Δ119-222) did not induce this redistribution of N (Supplementary Fig. 2B, C). These results suggest that residues 119–158, corresponding to the β1–β5 sheets of the CTD, are essential for N dephosphorylation. To further test this region, we generated an internal deletion mutant lacking residues 119-158 while preserving the remaining CTD (M-Δ119-158). This mutant failed to promote N dephosphorylation to the same extent as wild-type M (Fig. 3C, lane 17 vs. lane 16), supporting a role for residues 119–158 in the M–N interaction that facilitates N dephosphorylation. Consistently, the cryo-electron microscopy has shown that this region forms the primary structural cavity for N interaction [13]. Together, these data support a model in which N binding to M at ERGIC membrane is a prerequisite for its dephosphorylation.

We also observed different expression levels among the CTD-deleted mutants (Supplementary Fig. 3B). Cycloheximide-chase analysis further revealed differences in protein turnover among these constructs (Supplementary Fig. 3C-F), suggesting that residues 119–158 within the cytoplasmic region may also influence M protein stability.

## PP2A Phosphatase involved in the M-mediated N dephosphorylation and particle release

**Fig. 4:**
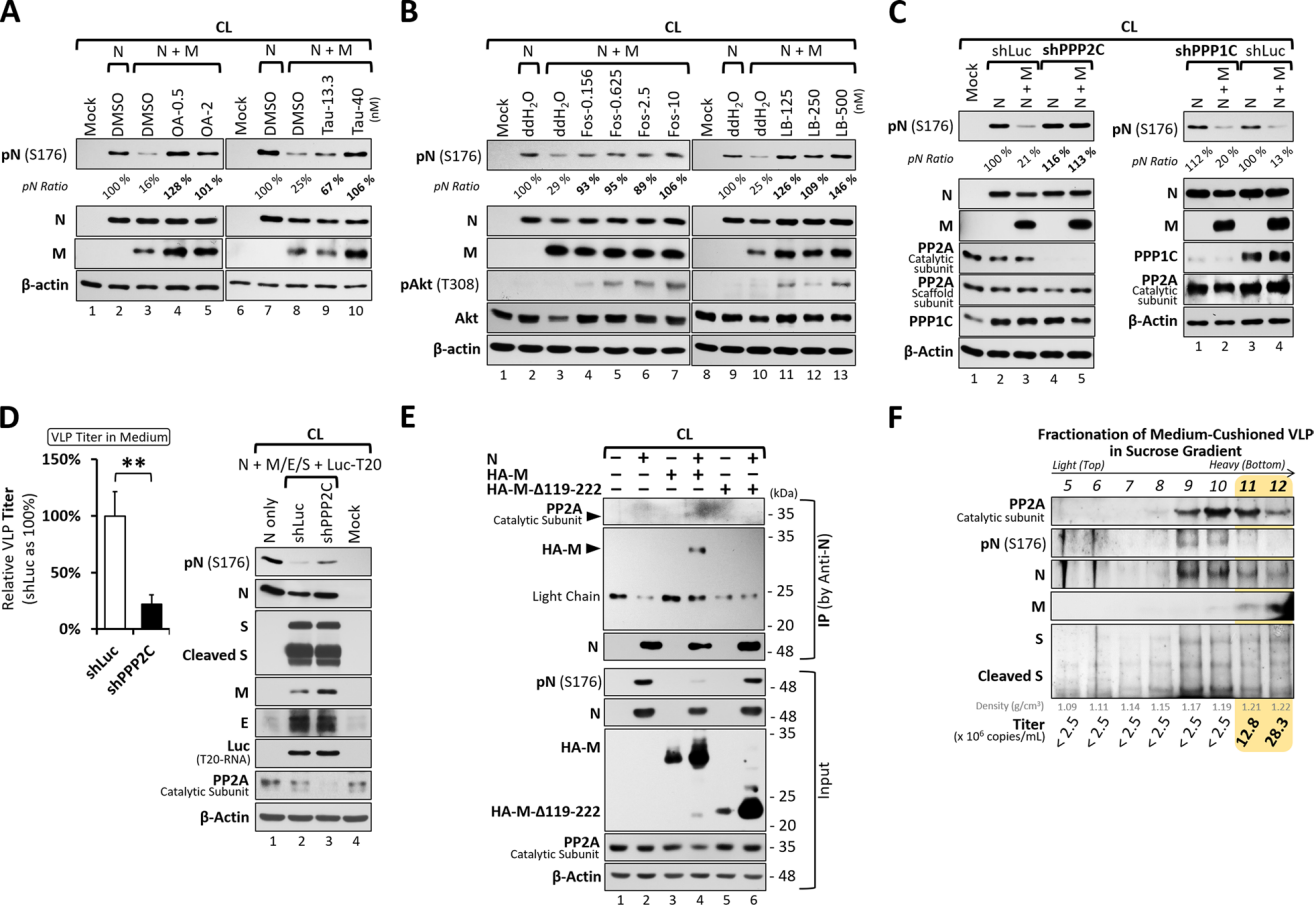
The Involvement of the Phosphatase PP2A in M-Mediated N Dephosphorylation and Particle Release.** A**, **B** Cell lysates from transfected 293T cells expressing the indicated viral proteins and treated with pan-PPase inhibitors (OA, okadaic acid; Tau, tautomycin) or PP2A-specific inhibitors (Fos, fostriecin; LB, LB-100) were analyzed by western blotting to evaluate the phosphorylation status of the N protein. **C** 293T cells co-expressing N and M proteins, with lentiviral knockdown of the catalytic subunit of PP2A (shPPP2C, left panels) or PP1 (shPPP1C, right panels), were lysed and analyzed by western blotting to assess N phosphorylation status. The pN ratios (phosphorylated N normalized to total N protein) were compared, with N expressed alone and the shLuc lentivirus-infected control set to 100%. **D** VLP titers in the culture media of producing 293T cells (co-transfected with N/M/E/S-expressing constructs and the packable T20-Luc RNA clone), with or without knockdown of the PP2A catalytic subunit (shPPP2C vs. shLuc control), were quantified by RT-qPCR. Data are presented as mean ± SD from three independent experiments (left bar graph). Cell lysates were analyzed in parallel by western blotting to confirm viral protein expression and knockdown efficiency (right panels). **p < 0.01. **E** The interaction of N and M proteins with the PP2A catalytic subunit was examined by co-immunoprecipitation using anti-N antibodies in lysates from 293T cells co-expressing N and M (48 h post-transfection). **F** Distribution of SARS-CoV-2 structural proteins (N, M, and S) and the PP2A catalytic subunit across fractions 5–12 was analyzed by western blotting. The yellow region indicates fractions 11–12 containing VLP-like particles, in which dephosphorylated N, PP2A, and T20-Luc RNA are detected. RNA titers and densities of the individual fractions are shown at the bottom of each lane

Given that the M proteins in the β-CoV lineage lack known phosphatase domains [19] and their N-terminal helices are embedded at ERGIC membrane, the β1–β5 sheets (residues 119–158) in its cytosolic CTD may serve as a molecular platform to recruit host PPases for N dephosphorylation. To test this hypothesis, two broad-spectrum phosphatase inhibitors, okadaic acid and tautomycin [28], were added to the VLP system. Upon treatment, the N protein was resistant to the M-induced dephosphorylation and remained phosphorylated (Fig. 4A, okadaic acid, lanes 4 and 5 vs. lane 3; tautomycin, lanes 9 and 10 vs. lane 8), suggesting that host phosphatases were involved in the N dephosphorylation process.

PP1 and PP2A are the predominant serine/threonine phosphatases in human cells, together accounting for over 90% of total activity [29, 30]. In the present study, we found that the concentration of okadaic acid required to block N dephosphorylation was ~ 0.5–2 nM (Fig. 4A, lanes 4 and 5), a range consistent with its IC₅₀ for PP2A (0.1–0.3 nM) but far lower than that for PP1 (15–50 nM) [28]. Furthermore, a recent interactome study identified PP2A as a putative interactor of the SARS-CoV-2 M protein [20]. Therefore, these findings imply that PP2A is the primary host phosphatase responsible for N dephosphorylation. To test this hypothesis, we examined the effects of the PP2A-selective inhibitors, fostriecin and LB-100 [28, 31], in the VLP system. At nanomolar concentrations well below their IC_50_ values for PP1 [28, 31], both inhibitors potently suppressed the M-induced N dephosphorylation (Fig. 4B; fostriecin, lanes 4–7 vs. lane 3; LB–100, lanes 11–13 vs. lane 10), implicating that PP2A is the principal host phosphatase mediating this process. By contrast, when the M protein was absent, inhibition of PP2A had no effect on the phosphorylation level of N protein, indicating that M is essential for initiating the PP2A-mediated N dephosphorylation (Supplementary Fig. 4).

To further validate these findings, lentiviral shRNAs were used to silence the catalytic subunits of PP2A (PPP2C) and PP1 (PPP1C) [32]. Only PPP2C knockdown, but not PPP1C, impaired M-induced N dephosphorylation (Fig. 4C, left panels, lanes 4–5 vs. 2–3; right panels, lanes 1–2 vs. 3–4). Because PP2A is a heterotrimer composed of scaffold, catalytic, and regulatory subunits, we also targeted the scaffold subunit with three independent shRNAs, which partially rescued N phosphorylation (Supplementary Fig. 5). Consistently, PPP2C knockdown reduced VLP titers by 80% (Fig. 4D), confirming that PP2A is critical for both M-mediated N dephosphorylation and VLP release.

To further characterize the M–N interaction, as well as its association with PP2A and their incorporation into VLPs, we performed CoIP and sucrose gradient fractionation analyses. Using lysates from 293T cells co-transfected with N and either wild-type M or the entire CTD-deleted mutant (M-Δ119-222), the CoIP data showed that N interacts with wild-type M but not the Δ119-222 mutant (Fig. 4E, lane 4 vs. lane 6). The IP complex also contained the catalytic subunit of PP2A and dephosphorylated N (Fig. 4E, lanes 4), indicating that the 119–222 segment of M is critical for N binding and recruitment of PP2A to facilitate N dephosphorylation.

Sucrose gradient analysis of cushioned culture media from cells co-expressing N, M, E, S, and a T20-Luc reporter revealed N protein across fractions 9–13 (Fig. 4F). Phosphorylated N was mainly detected in the lighter fractions (9–10) lacking M, suggesting its release independent of viral particles, whereas dephosphorylated N co-fractionated with M, PP2A catalytic subunit, and T20-Luc RNA in the heavier fractions (11–12), consistent with packaging into secreted particles. Although the apparent density distribution of the VLP-positive fractions differs from that reported previously [33], likely due to RNA packaging and prolonged centrifugation, this finding shows that the M protein co-fractionates with dephosphorylated N, PP2A, and T20-Luc RNA, supporting their association within VLP-like assemblies.

Together, these results support a model in which M-mediated N dephosphorylation via PP2A recruitment is linked to VLP assembly and release.

## M cooperates with N to recruit PP2A to ERGIC for N dephosphorylation

**Fig. 5:**
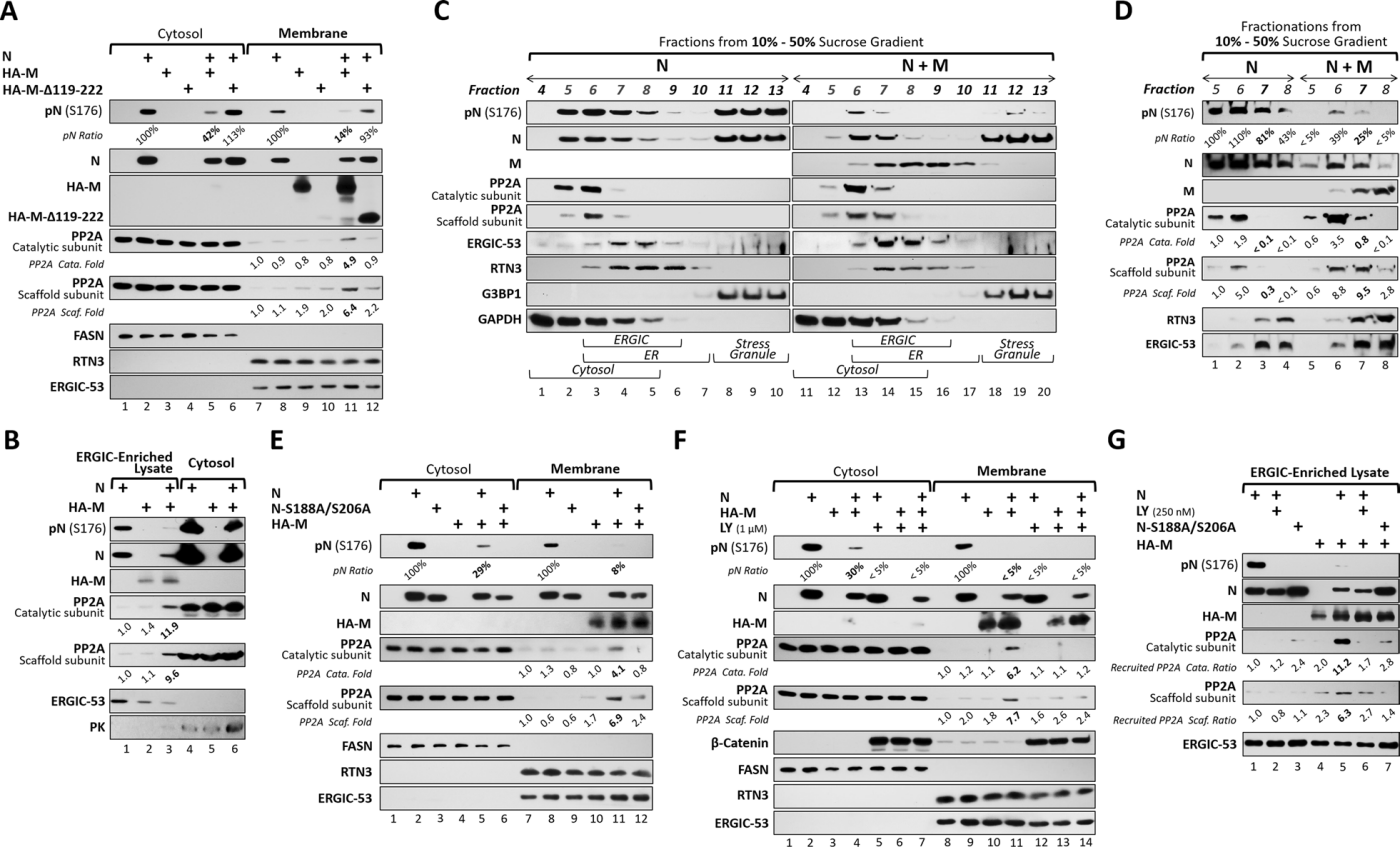
The M Protein Cooperates with Phosphorylated N to Recruit PP2A to the ERGIC for N Dephosphorylation.** A** 293T cells co-expressing N and M proteins were lysed for cytosol/membrane fractionation, followed by western blot analysis. **B** Isotonic lysates prepared from N/M-expressing 293T cells were subjected to sequential centrifugation at differential speeds. The final sedimented membrane fractions and concentrated supernatants were analyzed by western blotting. **C**, **D** N/M-expressing 293T cells were homogenized in isotonic buffer, and cell lysates were fractionated on a 10–50% sucrose gradient by ultracentrifugation. Equal volumes of fractions 4–13 were analyzed by western blotting (**C**). Similarly, equal volumes of ERGIC-containing fractions (F5–F8) obtained from lysates of N- or N/M-expressing 293T cells were analyzed side by side by western blotting (**D**). **E**, **F** Cytosol/membrane-fractionated lysates obtained from N/M-expressing 293T cells, either with expression of the phospho-mutant (N-S188A/S206A) (**E**) or following treatment with LY (1 μM) (**F**), were analyzed by western blotting. **G** As designed in (**E**) and (**F**), ERGIC-enriched lysates (i.e., the final sedimented membrane fraction obtained by sequential centrifugation) were analyzed by western blotting. ERGIC-53, RTN3, and G3BP1 served as subcellular markers for the ERGIC, ER lumen, and stress granules, respectively. FASN, PK, and GAPDH indicated cytosolic fractions. All blotting data are representative of three independent experiments

Cellular PP2A activity is predominantly localized in cytosol, with less than 10% associated with membrane compartments [34]. Transient recruitment of PP2A catalytic subunits to specific substrates via scaffold and regulatory proteins represents a key mechanism for regulating its phosphatase activity [34, 35]. To determine the subcellular site of M-mediated N dephosphorylation, we first performed cell fractionation to separate cell lysates into cytosolic and membrane fractions. Consistent with previous reports [36, 37], M was primarily detected in the membrane fraction (Fig. 5A, lane 9 vs. lane 3), N was distributed across both cytosolic and membrane fractions (Fig. 5A, lane 8 vs. lane 2), and the PP2A catalytic and scaffold subunits were enriched in the cytosolic fraction (Fig. 5A, lanes 1–6 vs. lanes 7–12). However, co-expression of M and N induced a marked redistribution of PP2A components, as both catalytic and scaffold subunits were accumulated in the membrane fraction, concomitantly with N dephosphorylation in this compartment (Fig. 5A, lane 11 vs. lane 8). By contrast, substitution of wild-type M with a CTD-deficient mutant (M-∆119-222) abrogated PP2A recruitment and preserved N phosphorylation state at the membrane fraction (Fig. 5A, lane 12 vs. lane 11). By IF staining, partial colocalization of PP2A with M-positive compartments is enhanced in the presence of both N and full-length M, whereas this effect is reduced with the M-Δ119-222 mutant (Supplementary Fig. 6A, B). Together, these findings demonstrate that M, via its CTD domain, cooperates with N to recruit PP2A to membrane compartments, thereby enabling N dephosphorylation.

Given that M is embedded in the ERGIC membrane [36], we next examined whether PP2A recruitment occurs specifically at this site. During early stage of CoV assembly, the interaction between M and N drives recruitment of the N-gRNA complex to M, facilitating genome packaging and virion maturation at ERGIC membranes [13, 36]. To assess whether the M-N complex mediates PP2A recruitment to the ERGIC, we performed differential centrifugation to isolate the ERGIC-enriched membrane fraction [25]. After sequential centrifugation, the fraction sedimented at 20,600 × *g* was enriched for ERGIC (marked by ERGIC-53), while the final supernatant contained cytosolic components (indicated by pyruvate kinase, PK) (Fig. 5B, lanes 1–3 vs. 4–6). Using this approach, co-expression of M and N, but not either protein alone, led to accumulation of PP2A components in the ERGIC-enriched fraction, coinciding with N dephosphorylation (Fig. 5B, lane 3 vs. lanes 1–2).

In addition, we applied sucrose density gradient analysis to further resolve distinct subcellular compartments [38]. In this assay, the ERGIC compartment localized predominantly to the separated fractions 7–8 (Fig. 5C, lanes 4–5 and 14–15, marked by ERGIC-53), whereas cytosolic proteins were enriched in the fractions 4–6 (Fig. 5C, lanes 1–3 and 11–13, marked by GAPDH). In the absence of M, N was broadly distributed across both cytosolic and membrane fractions and remained in a hyperphosphorylated state (Fig. 5C, lanes 2–5 and 8–10). By contrast, PP2A catalytic and scaffold subunits were largely restricted to cytosolic fractions (Fig. 5C, lanes 2 and 3). Upon M co-expression, the dispersed N in low-density fractions (5–8) became concentrated in the fractions 6–7 and converted to a hypophosphorylated state (Fig. 5C, lanes 12–15 vs. lanes 2–5). Concomitantly, PP2A catalytic and scaffold subunits were markedly enriched in the fraction 7, corresponding to the ERGIC compartment (Fig. 5C, lane 14 vs. lane 4). The G3BP1-containing fractions (11–13) mark stress granules and serve as a control, as only N, but not M or PP2A, was detected there, indicating that the N-M-PP2A complex specifically assembles in the ERGIC fractions (6–9). This redistribution was reproducibly observed, with representative fractions (5–8) from samples with or without M analyzed side by side, showing clear translocation of PP2A components to the ERGIC compartments (Fig. 5D, lane 7 vs. lane 3). Notably, the presence of packable T20-Luc RNA further enhanced the M-induced N dephosphorylation in membrane compartments (Supplementary Fig. 7A, lane 4 vs. lane 3; Supplementary Fig. 7B, lane 8 vs. lane 7), although it only modestly increased PP2A recruitment (Supplementary Fig. 7B, lane 8 vs. lane 7). Taken together, these results demonstrate that the ERGIC-localized M orchestrates the co-recruitment of N and PP2A to drive N dephosphorylation during virus assembly.

## Phosphorylation of N is required for PP2A recruitment

In virion morphogenesis, the SARS-CoV-2 N protein exists in two functionally distinct biomolecular states depending on its phosphorylation status. In its phosphorylated form, N exhibits liquid-like properties and associates with gRNA, loosening RNA secondary structures to facilitate viral transcription and replication [5, 6, 37]. Upon dephosphorylation, the gRNA-wrapped N proteins undergo phase transition and convert into gel-like condensates, which support viral genome packaging [15, 39]. However, whether the phosphorylation status of N serves as a prerequisite for recruiting host PP2A through the viral N–M complex remains to be determined.

To address this, we examined the necessity of N phosphorylation by using the phospho-deficient mutant or GSK-3 inhibitor. Compared with wild-type N, substitution with the S188A/S206A mutant markedly reduced recruitment of PP2A components to the membrane compartment (Fig. 5E, lane 12 vs. lane 11). Similarly, inhibition of GSK-3 activity by LY also impaired PP2A recruitment to the membrane (Fig. 5F, lane 14 vs. lane 11). These findings were further confirmed in ERGIC-enriched membrane fractions, either by the phospho-deficient mutant or using LY at 250 nM, a condition that maintains effective GSK-3 inhibition while minimizing potential perturbation of ERGIC and secretory organelle integrity (Fig. 5G, lanes 6 and 7 vs. lane 5). These results indicate that phosphorylated N not only serves as a substrate but also provides a prerequisite for efficient recruitment of PP2A subunits and subsequent dephosphorylation.

To assess the structural role of phosphorylated N in PP2A recruitment within the encapsidosome, we used AlphaFold 3.0 [40] to model the complexes containing either phosphorylated or non-phosphorylated N. Substitution of all serine/threonine residues in the SR region with alanine (N-15SA) induced an α-helical conformation in part of the central IDR (Supplementary Fig. 8A, graphic 3), resulting in the increase of hydrogen bonds that likely reduced structural flexibility and accessibility for complex formation [41]. In contrast, modeling of the S188A/S206A mutant showed a similarly flexible SR region as the wild-type and N-15SD proteins (Supplementary Fig. 8A, graphics 1, 2 and 4). Additional substitution of residues within the two phosphorylation stretches (N-10SA) also produced partial α-helical formation (Supplementary Fig. 8A, graphic 5), suggesting that reduced phosphorylation primed by S188 and S206 may shift the SR region toward a more rigid conformation.

In the presence of M, both wild-type N and the phospho-mimetic mutant (N-15SD) established close contacts with the CTD regions of the M dimer through their C-terminal IDRs and dimerization domains (Supplementary Fig. 8B, left and middle graphics), showing the consistency as previously reported [13, 15]. However, the phospho-deficient mutant (N-15SA) in the M-N complex exhibited a structural orientation shift, from the contact-exposed surface of the M dimer toward its ERGIC-embedded transmembrane domains (Supplementary Fig. 8B, right graphic vs. left and middle graphics). This structural protrusion may hinder the interaction of N-15SA with M and making it an unfavorable binding partner. By contrast, the phospho-mimetic N-15SD positioned its SR region within the M-N-PP2A complex to be accessible to the recruited PP2A catalytic subunit, with the central IDR located near the PP2A catalytic pocket (Supplementary Fig. 8C). This arrangement likely facilitates the presentation of phosphorylated residues to the PP2A active site, thereby promoting efficient N dephosphorylation. Notably, similar proximity of the central IDR to PP2A was also observed in other CoV N proteins with S-to-D substitutions (Supplementary Fig. 8D, 8E and 8F), highlighting the conserved requirement of PP2A for N dephosphorylation during CoV gRNA packaging and virion assembly.

## Blocking PP2A suppresses SARS-CoV-2 virion production

**Fig. 6:**
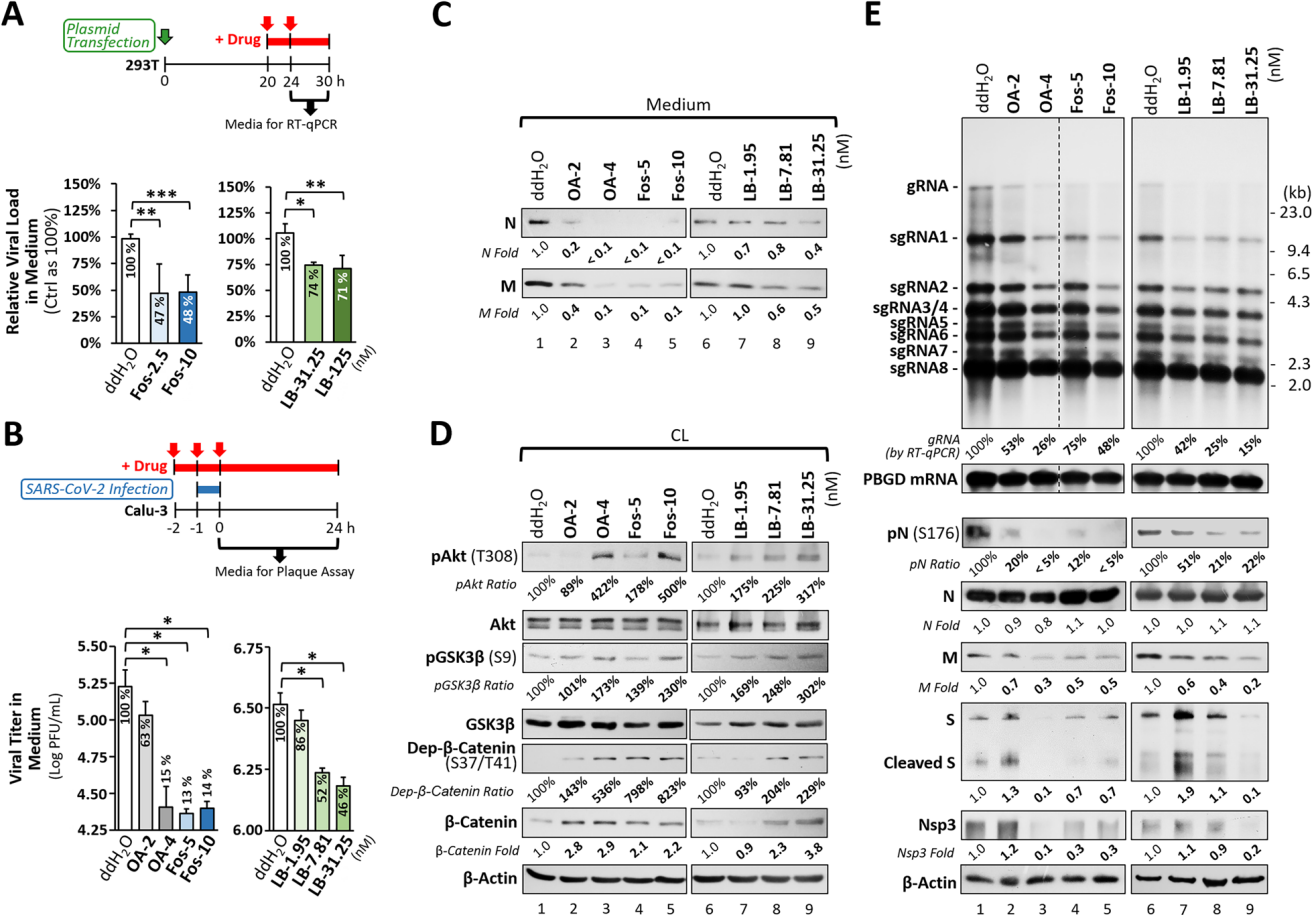
Blocking PP2A Activity Suppresses SARS-CoV-2 Virion Production and Decreases pgRNA Transcription.** A** Schematic illustration of the procedure used to examine the inhibitory effects of PP2A inhibitors on VLP release in the 293T culture system (upper diagram). VLP titers in culture supernatants collected from 293T cells treated with fostriecin (Fos) or LB-100 (LB) were determined by RT-qPCR. Data were obtained from three independent experiments and are presented as mean ± SD, with the ddH₂O-treated group set to 100% (lower bar graphs). **B** Similar to (**A**), a schematic of the protocol used to examine the antiviral effects of okadaic acid (OA) or PP2A inhibitors against SARS-CoV-2 infection in Calu-3 cells is shown (upper diagram). Culture supernatants collected at 24 h post-infection (MOI = 0.01) were analyzed by plaque assay to determine viral loads. Titers represent the average of three independent experiments and are shown as log₁₀ plaque-forming units (PFU)/mL, along with relative percentages compared to the ddH₂O-treated group (set to 100%) (lower bar graphs). **C** As described in (**B**), the corresponding culture supernatants were analyzed by western blotting to assess viral protein levels in released virions. **D**, **E** As described in (**C**), cell lysates and extracted RNAs from infected cells treated with OA or PP2A inhibitors were analyzed by western blotting and northern blotting, respectively, to assess Akt/GSK-3 signaling molecules (**D**), SARS-CoV-2 gRNA and individual sgRNAs (**E**, upper panels), and viral proteins (**E**, middle and lower panels). Relative gRNA levels determined by RT-qPCR (with PBGD as an internal control), relative levels of phosphorylated Akt, GSK-3, and N proteins (normalized to total protein), and fold changes in viral N, M, S, and Nsp3 proteins were compared with the ddH₂O-treated group (set to 1.0) and shown below. All blotting data are representative of three independent experiments. *p < 0.05; **p < 0.01; ***p < 0.001

Given the critical role of PP2A in N dephosphorylation during viral assembly, we hypothesized that inhibiting PP2A activity would suppress SARS-CoV-2 virion production. To test this, we applied PP2A inhibitors in the 293T-based VLP system. At 20 h post-transfection, cells were treated with increasing doses of fostriecin or LB-100 for 10 h. VLP secretion, quantified by RT-qPCR of packaged T20-Luc RNAs in culture supernatants (collected from the final 6 h), was significantly reduced by both inhibitors compared to the controls (Fig. 6A), indicating that PP2A activity is required for efficient VLP release.

We next evaluated the antiviral efficacy of PP2A inhibitors in Calu-3 cells infected with the SARS-CoV-2 Omicron BA.2 variant under BSL-3 conditions. Cells were pretreated with okadaic acid, fostriecin, or LB-100 for 1 h prior to infection, and treatment was maintained throughout the experiment. At 24 h post-infection, the viral titers in supernatants measured by plaque assay revealed that okadaic acid and fostriecin reduced virus production by ~ 85%, while LB-100 caused a 54% reduction (Fig. 6B). Consistently, the levels of N and M proteins from secreted virions in supernatants were markedly diminished following inhibitor treatment (Fig. 6C, lanes 2–5 vs. lane 1; lanes 7–9 vs. lane 6). Comparable antiviral effects were also observed against the Omicron subvariant XBB.1.16 [1] (Supplementary Fig. 9), suggesting that PP2A inhibition broadly suppresses SARS-CoV-2 progeny production. Moreover, combining LB-100 with camostat, a known viral entry blocker [42], exhibited an additive inhibitory effect (Supplementary Fig. 10, bars 7–8 vs. 3–4), highlighting the potential of PP2A inhibitors as part of combination antiviral strategies.

From a cellular signaling perspective, PP2A inhibition indirectly activates Akt by maintaining the phosphorylation level at its T308 site, a known PP2A target [43]. Then, the activated Akt subsequently suppresses GSK-3 activity through phosphorylating the inhibitory S9 site [43]. Given that GSK-3 is a major kinase responsible for N phosphorylation [8], its decreased activity is expected to reduce the phosphorylated level of N. Consistently, treatment with okadaic acid or other PP2A inhibitors altered the Akt/GSK-3 signaling axis, as indicated by increased phosphorylation of Akt (T308) and GSK-3 (S9), as well as accumulation of dephosphorylated β-catenin (S37/T41) and total β-catenin, a GSK-3 substrate, reflecting suppressed GSK-3 activity (Fig. 6D, lanes 2–5 vs. lane 1; lanes 7–9 vs. lane 6). These effects were evident in virus-infected Calu-3 cells but not in 293T cells, which exhibit constitutively high Akt activity due to SV40 large T antigen expression [44] (Supplementary Fig. 4). Consequently, PP2A inhibition resulted in the decreased N phosphorylation in virus-infected cells (Fig. 6E, middle panels, lanes 2–5 vs. lane 1; lanes 7–9 vs. lane 6).

Our previous studies demonstrated that the GSK-3-mediated N phosphorylation is essential for continuous transcription of SARS-CoV-1 [5]. Unsurprisingly, following PP2A inhibitor treatment, the reduced N phosphorylation correlated with the decrease of gRNA and longer sgRNAs generated via continuous transcription, as detected by Northern blot and RT-qPCR (Fig. 6E, upper panels, lanes 2–5 vs. lane 1; lanes 7–9 vs. lane 6). Immunoblot analysis further confirmed the reduced expression of gRNA-encoded Nsp3 and longer sgRNA-encoded S and M proteins, whereas the N protein, encoded by the shortest sgRNA, remained largely unaffected (Fig. 6E, lower panels, lanes 2–5 vs. lane 1; lanes 7–9 vs. lane 6).

Together, these findings indicate that PP2A inhibition disrupts two critical stages of the CoV life cycle regulated by N phosphorylation and dephosphorylation: continuous transcription of viral gRNA and longer sgRNAs, and assembly and release of mature virions. By targeting these processes, PP2A inhibitors exert potent anti-SARS-CoV-2 activity. Notably, these inhibitors also display broad-spectrum antiviral effects against other coronaviruses, including JHMV (a lineage A β-CoV) and human CoV 229E (an alpha-coronavirus) [45] (Supplementary Fig. 11).

## Discussion

Our previous studies on SARS-CoV-1 and JHMV first identified the major phosphorylation sites in the central SR-rich motif of the CoV N protein, with GSK-3 as the key responsible kinase [5, 6]. Notably, we observed a dynamic shift of viral N protein from the highly phosphorylation form after its newly synthesis in cytosol to a hypophosphorylated status in secreted mature virions [5, 6]. In the current study, we showed that this transition also occurs in SARS-CoV-2. Furthermore, we revealed that the phosphorylation-to-dephosphorylation switch during the late stage of viral life cycle is mediated by the M–N–dependent recruitment of host PP2A to the ERGIC membrane.

As shown in our and others’ previous work, phosphorylation of CoV N protein in early replicative stage is important for gRNA and longer sgRNAs synthesis [5, 6, 9, 46]. Here, focusing on the late stage, we found that PP2A-mediated N dephosphorylation is essential for complete viral genome packaging and subsequent release of mature virions. Consistently, a recent genome-wide CRISPR knockout screen supports the critical role of PP2A in the SARS-CoV-2 replication cycle, showing that the genes encoding PP2A catalytic and regulatory subunits, but not PP1 components, significantly enhance viral infection [47]. Together, our findings highlight the involvement of host PP2A in coronavirus progeny production and virion morphogenesis, emphasizing its essential role in the life cycle of SARS-CoV-2 and potentially other CoVs.

In the CoV life cycle, phosphorylated and non-phosphorylated N proteins perform distinct roles at different stages. Following viral entry, the newly synthesized N proteins, which are highly phosphorylated by host kinases, act as liquid-like partners to facilitate continuous transcription of sgRNAs and gRNA [5, 6]. After replication, the M protein at ERGIC membrane functions as a hub by interacting with the PS of gRNA, as well as N proteins, which wrap the viral genome like beads on a string for initiation of packaging [36]. Within this encapsidosome, we found that the N/M complex (particularly the phosphorylated N) facilitates the association with PP2A, thereby promoting N dephosphorylation. This finding is supported by complementary approaches, including CoIP, mutational analyses, sucrose gradient fractionation, and IF imaging (Fig. 4E, F; Supplementary Fig. 6). This transition converts the N-gRNA complex from flexible strings to gel-like condensates, promoting viral genome packaging into assembled virions for subsequent release. Notably, this phosphorylation-to-dephosphorylation switch of capsid proteins appears broadly conserved during virion assembly in other viruses, such as hepatitis B virus [10], West Nile virus [11], and rubella virus [12]. Whether this dynamic transition is universal among DNA and RNA viruses remains to be explored.

The PP2A core enzyme is a heterodimer composed of a catalytic subunit and a scaffold subunit. Association with a regulatory subunit forms the trimeric PP2A holoenzyme, which exhibits phosphatase activity. The specific regulatory subunit largely determines the repertoire of PP2A substrates and their subcellular localization [48]. Interestingly, some viruses hijack host PP2A to redirect its PPase activity for supporting their life cycles. For example, the adenovirus E4orf4 protein interacts with PP2A components to mediate dephosphorylation of viral E1A and host transcription factor c-Fos, facilitating the transition from early to late viral gene expression [49]. Similarly, the secreted Vpr protein of HIV-1 binds host PP2A, potentially modulating its activity to induce T-cell apoptosis and promote immune evasion [50]. In SARS-CoV-2, we found that the M protein dimer at the ERGIC membrane may mimic a substrate-recognizing regulatory subunit by binding phosphorylated N protein, thereby recruiting the PP2A core enzyme to catalyze N dephosphorylation. Importantly, M is indispensable for this process, as PP2A inhibition alone does not increase N phosphorylation in its absence (Supplementary Fig. 4). Whether other PP2A regulatory subunits participate in this process remains to be investigated.

Considering the critical role of N phosphorylation and dephosphorylation in the CoV replication cycle, both the previously identified GSK-3 kinase [5, 6, 8] and the newly defined PP2A represent potential targets for development of antiviral compounds. Indeed, GSK-3 inhibitors such as CHIR-99021 and the nonspecific inhibitor kenpaullone have been reported to reduce SARS-CoV-2 viral loads by ~ 50–90% at micromolar (μM) concentrations in cultured cells [8, 51]. However, the broad physiological functions of GSK-3 limit the therapeutic application of its inhibitors due to significant toxicity, particularly because the effective dosages of most small-molecule inhibitors in phase I or II clinical trials are typically in μM range [52, 53]. For instance, lithium, an FDA-approved GSK-3 inhibitor, achieves therapeutic effects at ~ 0.6 mM for bipolar disorder [54], far above the concentrations typically used for antiviral studies. Moreover, we observed that GSK-3 inhibition paradoxically increased the secretion of VLPs (Fig. 2B, D). This finding raises an important concern that incomplete suppression of N phosphorylation at tolerable inhibitor doses may inadvertently enhance viral spread.

In contrast, pharmacological blockade of PP2A may represent a novel antiviral strategy against SARS-CoV-2 by interfering with virion maturation and egress during the late stage of CoV life cycle. The PI3K/Akt pathway is exploited by viruses to support replication. Akt inhibitors such as capivasertib and MK-2206 reduce SARS-CoV-2 entry and viral RNA/virion production in vitro [55, 56]. However, Akt negatively regulates GSK-3, which is an essential kinase to phosphorylate the coronavirus N protein [5, 9], so Akt inhibition may have context-dependent effects on viral replication. Notably, inhibition of cellular PP2A also suppresses GSK-3 activity through sustained Akt activation [43]. Hence, this cross-regulatory interaction impairs phosphorylation of newly synthesized N proteins, thereby reducing gRNA transcription and progeny virus production via an Akt/GSK-3 feedback loop (Fig. 6D, E). In our present study, the PP2A inhibitor LB-100, a compound currently in phase 2 clinical trials [57], demonstrated potent antiviral activity at nanomolar concentrations (Fig. 6B). Moreover, LB-100 showed additive effects with the entry blocker camostat [42], achieving a 1.0-log reduction in viral load (Supplementary Fig. 10), and displayed broad-spectrum antiviral activity against JHMV and HCoV-229E (Supplementary Fig. 11). Further validation of the antiviral efficacy of the PP2A inhibitors in preclinical animal models is warranted to be evaluated for drug development.

## Conclusions

Taken together, our study confirmed that the dynamic phosphorylation change of SARS-CoV-2 N protein at different stages of life cycle, as previously found in other CoVs. The viral M protein orchestrates the phosphorylated N to recruits PP2A to ERGIC for N dephosphorylation, which enhances virion assembly and release. Given the critical role of host PP2A phosphatase in regulating progeny virion production, targeting PP2A, either alone or in combination with existing therapies, may offer a promising antiviral strategy against SARS-CoV-2 and potentially other CoVs.

## Supplementary Information

Supplementary File 1.

## Data Availability

All the data and materials that support the findings of this study are available from the corresponding author upon reasonable request.

## Abbreviations

N: Viral nucleocapsid protein
SARS-CoV-1: Severe acute respiratory syndrome coronavirus 1
JHMV: The JHM strain of mouse hepatitis virus
SARS-CoV-2: Severe acute respiratory syndrome coronavirus 2
M: Viral membrane protein
E: Viral envelope protein
S: Viral spike protein
PP2A: Protein phosphatase 2A
VLP: Virus-like particle
ERGIC: Endoplasmic reticulum-Golgi intermediate compartment
GSK-3: Glycogen synthase kinase-3
