# Supplementary material for: SARS-CoV-2 membrane protein recruits PP2A to dephosphorylate the nucleocapsid and promote virion production
Source: J Biomed Sci. 2026 May 15;33:51. doi: 10.1186/s12929-026-01255-w (PMC13179615; doi:10.1186/s12929-026-01255-w)

**Supplemental Information**

**SARS-CoV-2 Membrane Protein Recruits PP2A to Dephosphorylate the Nucleocapsid and Promote Virion Production**

**Authors**

Sheng-Han Wang, Tai-Ling Chao, Chi-Ling Hsieh, Pei-Jer Chen, Sui-Yuan Chang, and Shiou-Hwei Yeh

**Supplementary Materials and Methods**

***Transmission Electron Microscopy***

The VLP precipitates prepared from cushioned media as described in the main text were fixed by adding a mixture of glutaraldehyde and paraformaldehyde to final concentrations of 2% and 2%, respectively, and incubated overnight at 4°C to preserve particle morphology. An aliquot of the fixed sample was then applied onto carbon-coated copper grids and allowed to adsorb for several minutes. Excess liquid was removed with filter paper, and the grids were negatively stained with 1–2% Uranyl acetate to enhance contrast. After air drying, the grids were examined using a transmission electron microscope (TEM) operated at an appropriate accelerating voltage. Micrographs of VLPs were acquired to assess the morphology and approximate size distribution.

**Immunofluorescence Staining**

For immunofluorescence (IF) staining, the Huh7 cells cultured on glass coverslips and transfected with plasmids encoding the viral N/M proteins (a molar ratio of 1:3) at 30 or 48 hrs post-transfection were washed with phosphate buffered saline (PBS) and fixed with 4% paraformaldehyde for 10 min at room temperature. After fixation, cells were permeabilized with 0.2% Triton X-100 in PBS for 15 min and subsequently blocked with 5% fetal bovine serum plus 3% bovine serum albumin in PBS for 1 hr to reduce nonspecific binding. Thereafter, cells were then incubated with primary antibodies in blocking buffer for overnight at 4 °C. After washing with PBS, cells were incubated with fluorophore-conjugated secondary antibodies for 1 hr at room temperature in the dark, and nuclei were counterstained by 4’,6-diamidino-2-phenylindole. Finally, coverslips were mounted onto glass slides and images were acquired by confocal microscope. The percentage of cells showing yellow signals, which indicate the colocalization of N or the PP2A catalytic subunit (red fluorescence) with M (green fluorescence), was quantified (n > 30 cells) and analyzed using the chi-square test. Cells displaying abnormal morphology, including shrunken or deformed nuclei compared with mock controls, were excluded to ensure data quality.

***Prediction of Protein Complex Model***

To analyze the structural details in the M-N-PP2A complex, the amino acid sequences of SARS-CoV-2 M protein (#P0DTC5), N protein (#P0DTC9), PP2A catalytic (#B3KQ51) and scaffold subunits (#C9J9C1), all obtained from Uniprot, were used for prediction by Alphafold 3.0. In addition, the N-15SD, N-15SA, N-S188A/S206A or N-10SA mutant, which has aspartates or alanines substituted for the corresponding serine/threonine residues in the SR-rich region (amino acid 176–206) of N protein, were included for analysis. The SARS-CoV-1 M (#P59596) and N proteins (#P59595), the JHMV M (#P08549) and N proteins (#P03417), as well as the HCoV-229E M (#A0A1L7B920) and N proteins (#P15130), were also input to predict the virus-specific complex models. Similar to the N-15SD mutant of SARS-CoV-2, the phospho-mimetic ones of SARS-CoV-1 N [N-14SD, aspartate residues substituted for all 14 serine/threonine residues in the SR-rich region (amino acid 177–207)], JHMV-N [N-4SD, aspartate residues substituted for all 4 serine residues in the central IDR region (amino acid 197–209)] and HCoV-229E-N [N-4SD, aspartate residues substituted for all 4 serine residues in the central IDR region (amino acid 149–161)] were replaced for their wild-type forms, respectively. For JHMV-M/N protein docking, the mouse-originated PP2A scaffold (#Q76MZ3) and catalytic subunits (#P63330) were also applied. The top ranked models of each cases obtained from the Alphafold-predicted pools were visually analyzed by ChimeraX (UCSF, version 1.8).

**Supplementary Figures**

**
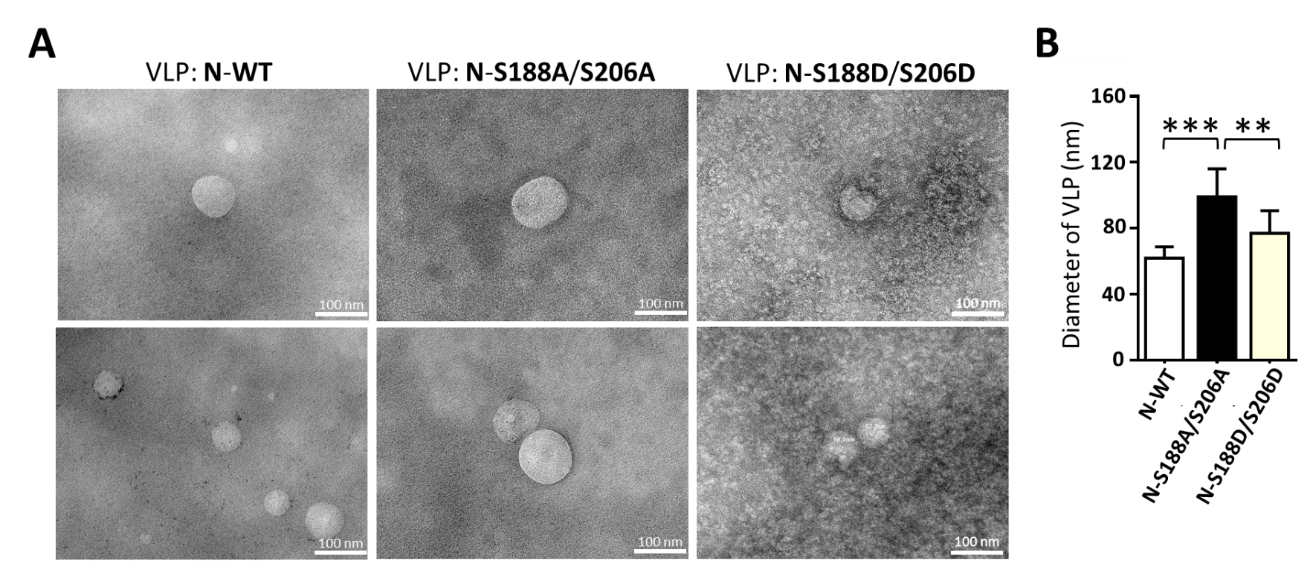
Supplementary Figure 1**

**Supplementary Figure 1. Morphological and Functional Evaluation of SARS-CoV-2 VLPs. (A)** Representative transmission electron microscopy images of SARS-CoV-2 VLPs containing either wild-type (WT), phospho-deficient (S188A/S206A), or phospho-mimetic (S188D/S206D) N proteins. **(B)** The diameters of these VLPs were quantified by measuring >30 particles in each group. Scale bar, 100 nm. **p < 0.01; ***p < 0001.

**Supplementary Figure 2**

**
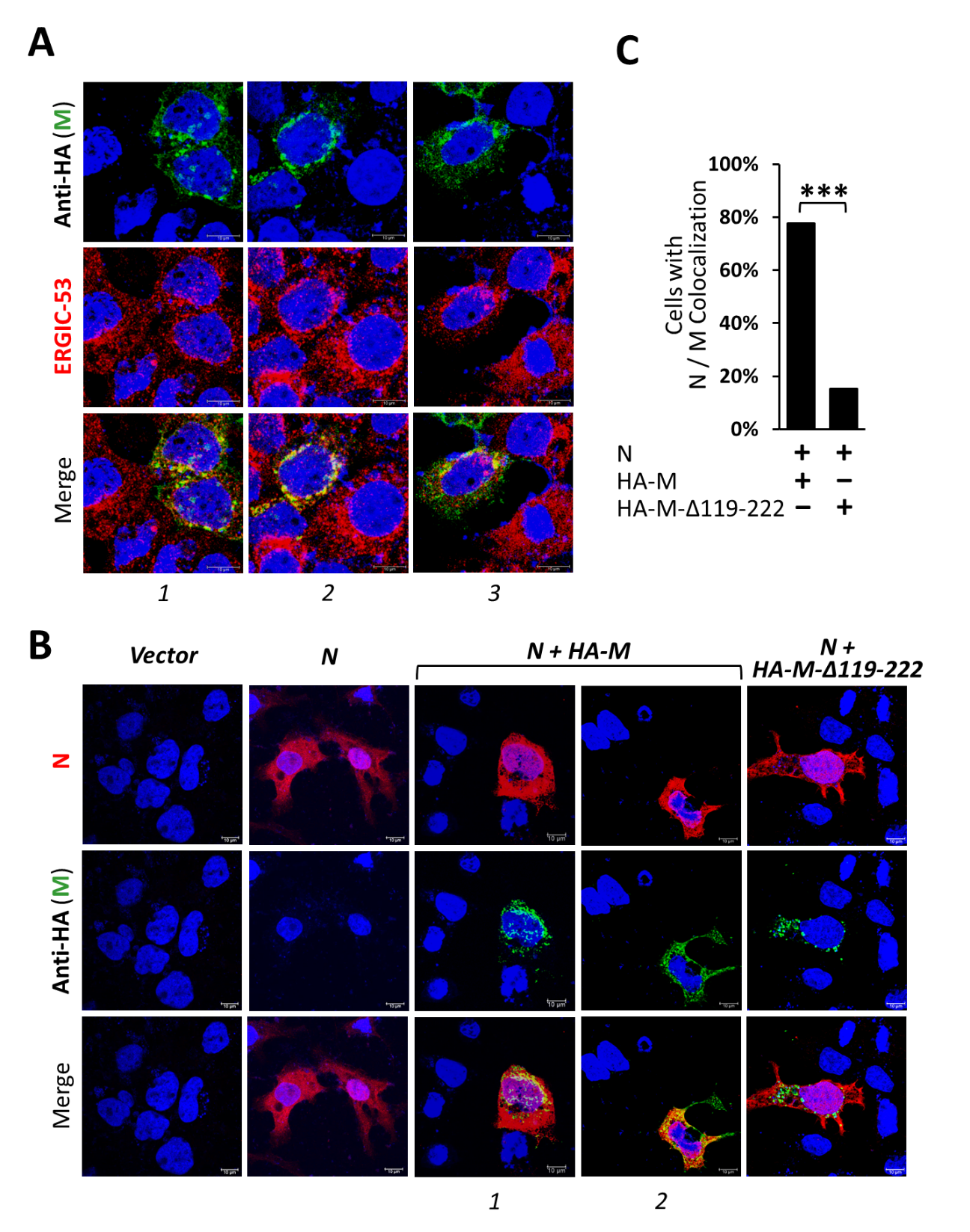
**

**Supplementary Figure 2. Immunofluorescence Staining of N and M Proteins.** **(A)** Huh7 cells expressing M protein alone were fixed at 30 h post-transfection and subjected to immunofluorescence staining for M and ERGIC-53. **(B)** Huh7 cells co-expressing N and M proteins (transfected at a molar ratio of 1:3) were fixed at 48 h post-transfection and stained for N and M. Yellow signals indicate colocalization of the N and M proteins. **(C)** The percentage of cells showing colocalization of N (red) and M (green) was quantified (n > 30 cells) and statistically analyzed using the chi-square test. Scale bar, 10 μm. ***p < 0.001.

**
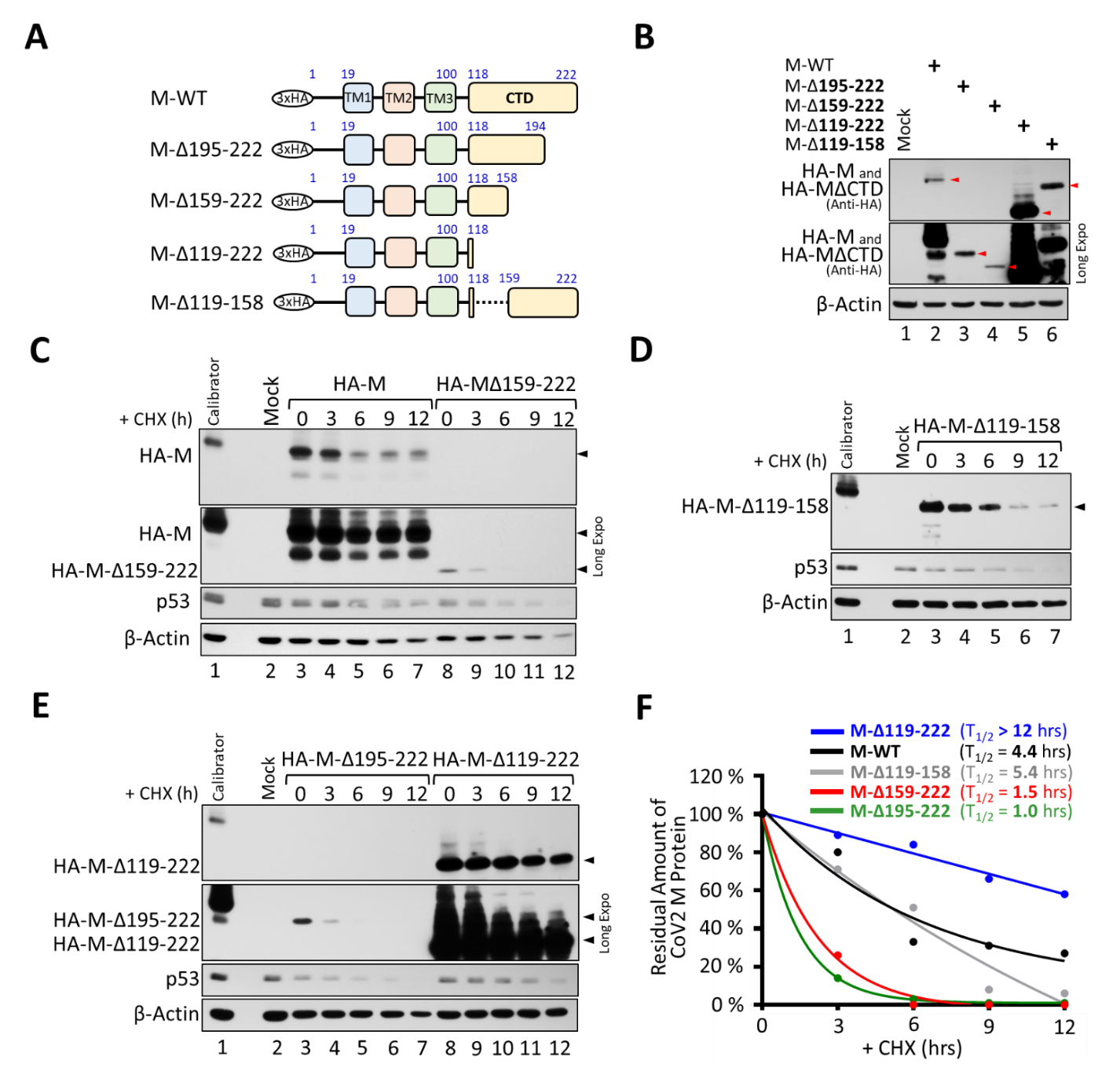
Supplementary Figure 3**

**Supplementary Figure 3. Evaluation of the Expression Level and Half-Life of M Protein.** **(A)** Schematic illustration of HA-tagged constructs expressing M proteins with serial C-terminal truncations or internal deletions. The numbers above the diagrams indicate the amino acid residues of the M protein. **(B)** Cell lysates prepared from 293T cells transfected with M-expressing constructs (at equal molar ratios) were analyzed by western blotting to evaluate protein expression levels. **(C–E)** As described in (B), transfected cells were treated with cycloheximide (CHX) at 48 h post-transfection, lysed at the indicated time points, and analyzed by western blotting. **(F)** Based on these results, decay curves for the M protein and its deletion mutants were generated to determine their respective half-lives. Data are representative of two independent experiments.

**Supplementary Figure 4**

**
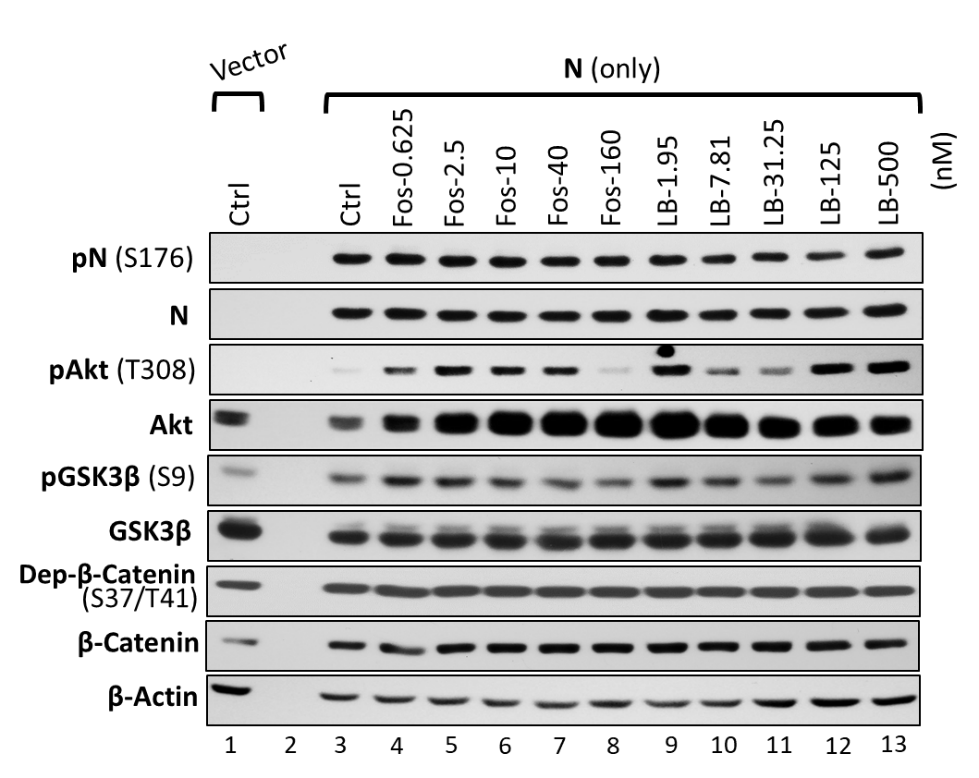
**

**Supplementary Figure 4. Inhibition of PP2A Does Not Influence N Phosphorylation or the Akt-GSK-3 Pathway in 293T Cells.** 293T cells transfected with the N-expressing plasmids were treated with PP2A-specific inhibitors (Fos, fostriecin; LB, LB-100) for 24 h and then lysed to examine N phosphorylation levels and Akt–GSK-3 pathway activity by western blotting. Data are representative of two independent experiments.

**Supplementary Figure 5**

**
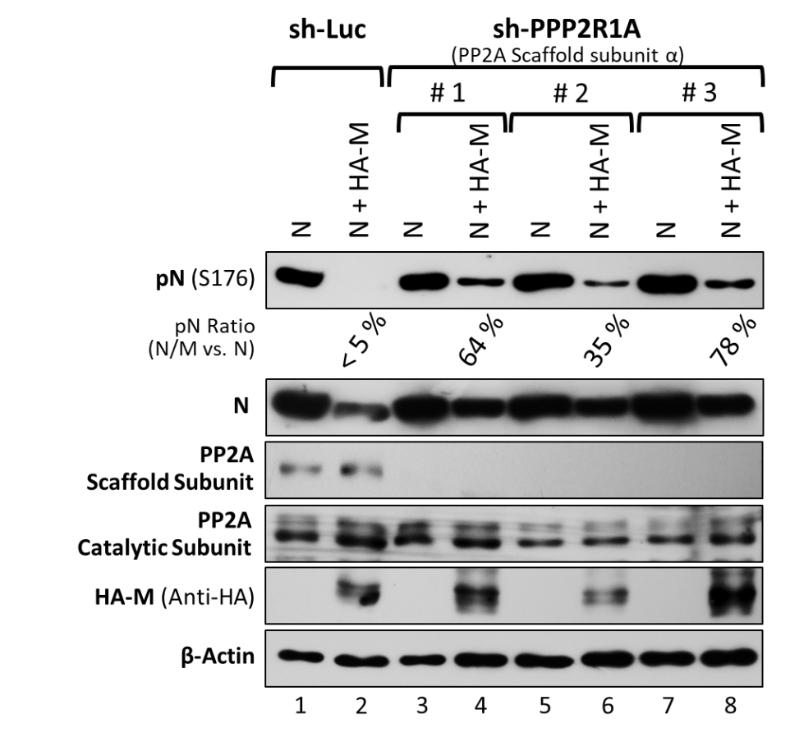
**

**Supplementary Figure 5. The PP2A Scaffold Subunit is Important for M-Induced N Dephosphorylation.** Cell lysates from 293T cells co-expressing N and M, with or without knockdown of the PP2A scaffold subunit α (shPPP2R1A; shLuc as control), were analyzed by western blotting to evaluate the ability of the M protein to induce N dephosphorylation. Data are representative of two independent experiments.

**
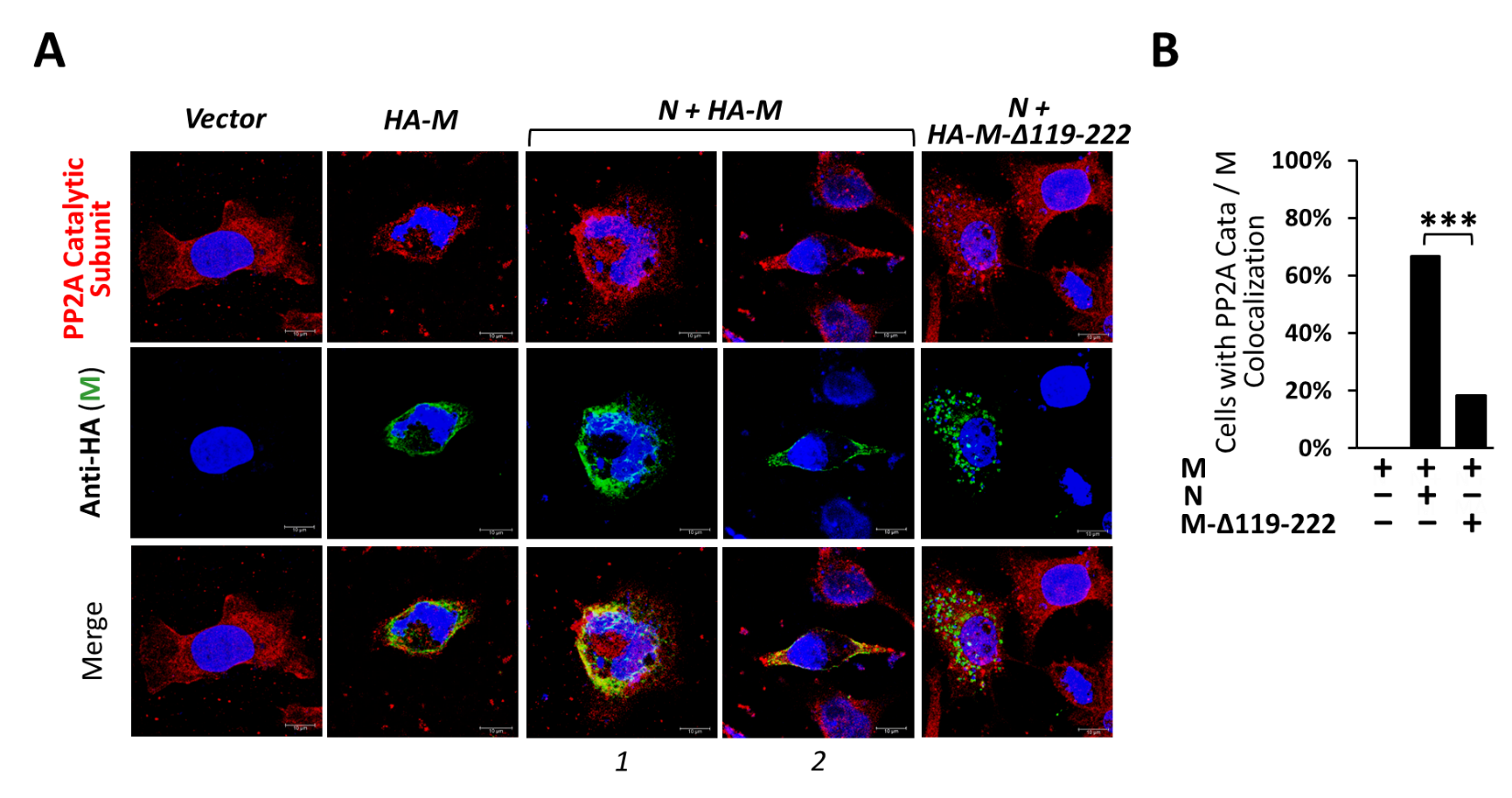
Supplementary Figure 6**

**Supplementary Figure 6. Immunofluorescence Staining of PP2A Catalytic Subunit and M Protein.** **(A)** Huh7 cells co-expressing N and M proteins (transfected at a molar ratio of 1:3) were fixed at 48 h post-transfection and subjected to immunofluorescence staining for PP2A catalytic subunit and M protein. Yellow signals indicate colocalization of PP2A and M protein. **(B)** The percentage of cells showing colocalization of PP2A catalytic subunit (red) with M protein (green) was quantified (n > 30 cells) and statistically analyzed using the chi-square test. Scale bar, 10 μm. ***p < 0.001.

**Supplementary Figure 7**

**
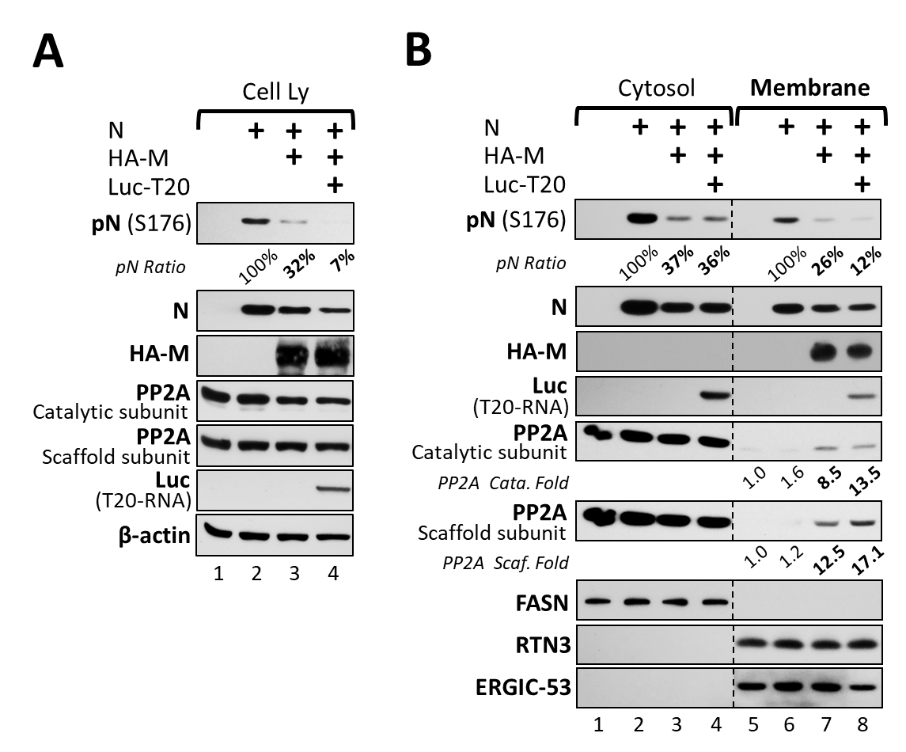
**

**Supplementary Figure 7. Packaged T20-Luc RNA Enhanced M-Induced N Dephosphorylation.** 293T cells cotransfected with N- and M-expressing plasmids, along with the T20-Luc construct, were lysed at 48 h post-transfection to obtain crude cell lysates (A) or cytosolic and membrane fractions (B). The samples were then subjected to electrophoresis and western blotting to evaluate N phosphorylation and PP2A recruitment. The relative amounts of PP2A catalytic and scaffold subunits in the membrane-fractionated lysates were compared and normalized to the respective ERGIC-53 levels, with the mock control set to 1.0. Data are representative of three independent experiments.

**
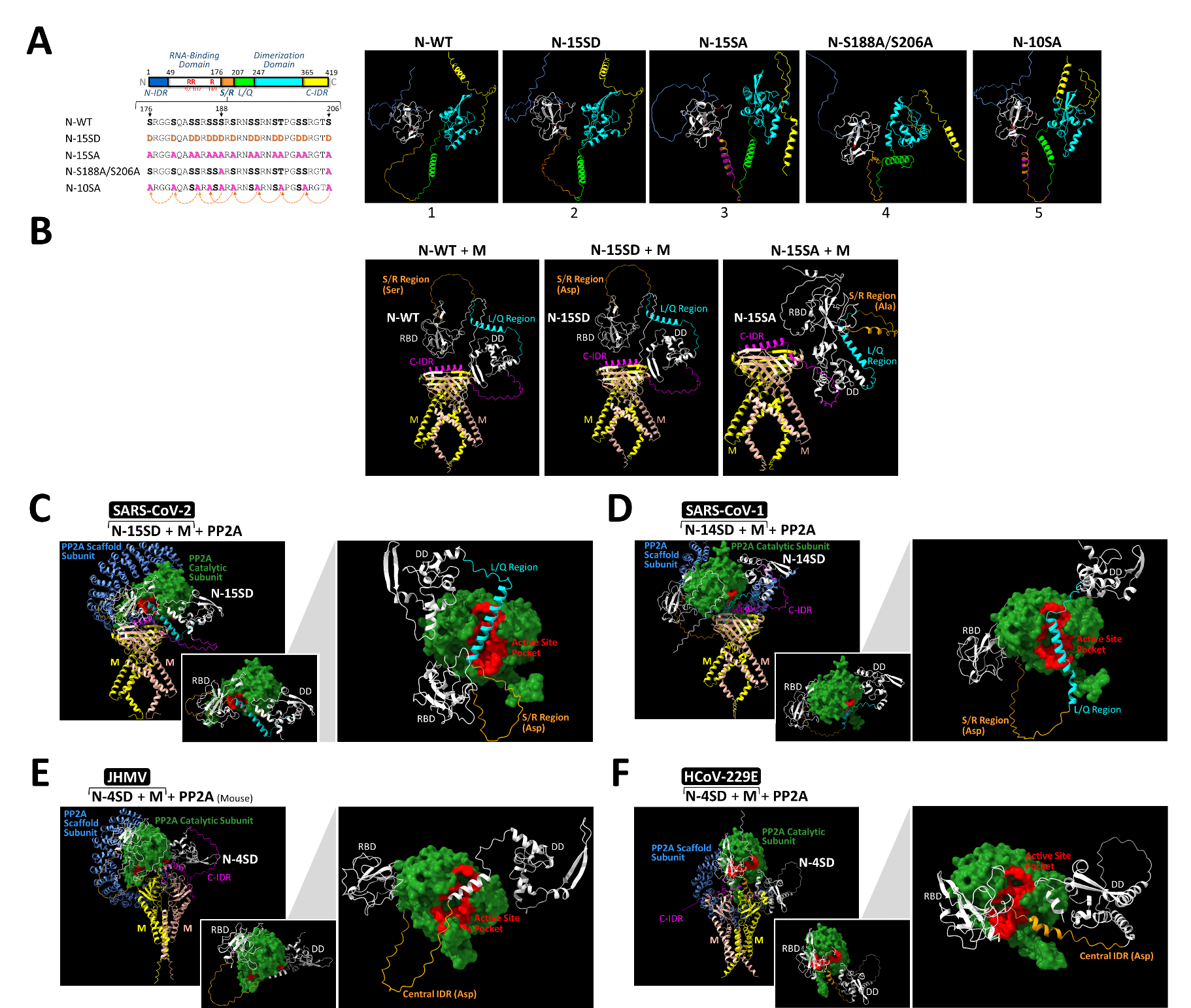
Supplementary Figure 8**

**Supplementary Figure 8. Structural Comparison of Coronavirus N Proteins with Distinct Phospho-Mimetic States in Viral M-N Complexex with Host PP2A. (A)** Schematic illustration of the functional domains of the SARS-CoV-2 N protein. The corresponding amino acid positions and key arginine residues (shown in red) responsible for viral RNA binding are indicated (left diagram). The 15 serine/threonine residues within the SR-rich region of the N protein were substituted with either aspartates or alanines to generate phospho-mimetic (N-15SD) or phospho-deficient (N-15SA) mutants, respectively. In addition, a priming-defective mutant with S-to-A substitutions at Ser188 and Ser206 (N-S188A/S206A), and a downstream phosphorylation-deficient mutant (N-10SA), were also included. These mutants, along with wild-type N (N-WT), were subjected to structural comparison using AlphaFold 3.0 (right panels). N-IDR, N-terminal intrinsically disordered region; RBD, RNA-binding domain; DD, dimerization domain; C-IDR, C-terminal intrinsically disordered region. **(B)** As in described in (A), structural differences among SARS-CoV-2 complexes containing the M dimer and N protein (WT, N-15SD, or N-15SA) were evaluated. **(C)** The docking model of the SARS-CoV-2 M–N(15SD)–PP2A complex predicted by AlphaFold 3.0 is shown (left panel). The PP2A catalytic subunit (shown in forest green) is highlighted by its molecular surface, with residues in the active-site pocket marked in red. The inset (lower right panel) shows a simplified contact model between the PP2A catalytic subunit and the N protein with a concealed C-IDR. An enlarged view with an adjusted perspective is also shown (right panel) to highlight the proximity of the SR-rich region (orange) of the N protein to the active-site pocket of the PP2A catalytic subunit (red). **(D–F)** As described in (C), M–N(SD)–PP2A complex models from SARS-CoV-1 (D), JHMV (E), and HCoV-229E (F) were predicted using AlphaFold 3.0 for comparison as well.

**Supplementary Figure 9**


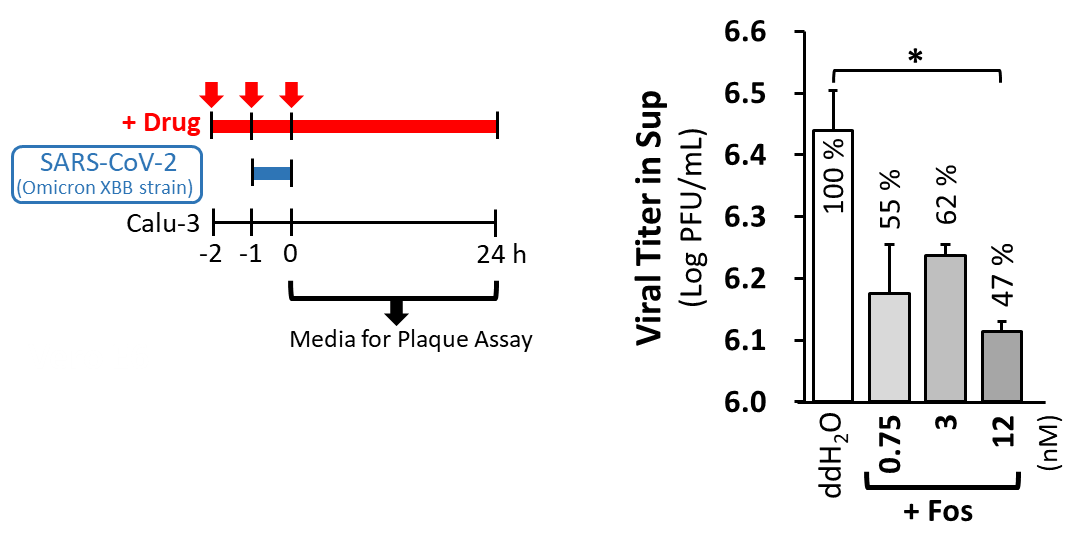


**Supplementary Figure 9. Inhibition of PP2A Activity Reduces the Viral Load of the Omicron XBB Subvariant.** The experimental workflow for evaluating the antiviral effect of fostriecin (Fos) against the Omicron XBB subvariant of SARS-CoV-2 in infected Calu-3 cells is schematically illustrated (left diagram). Culture supernatants were collected and analyzed by plaque assay to determine viral load. Viral titers from two independent experiments are presented as log values (mean ± SD) of plaque-forming units (PFU) per mL, along with relative percentages compared with the ddH₂O control group (set to 100%) (right bar graph). *p < 0.05.

**Supplementary Figure 10**

**
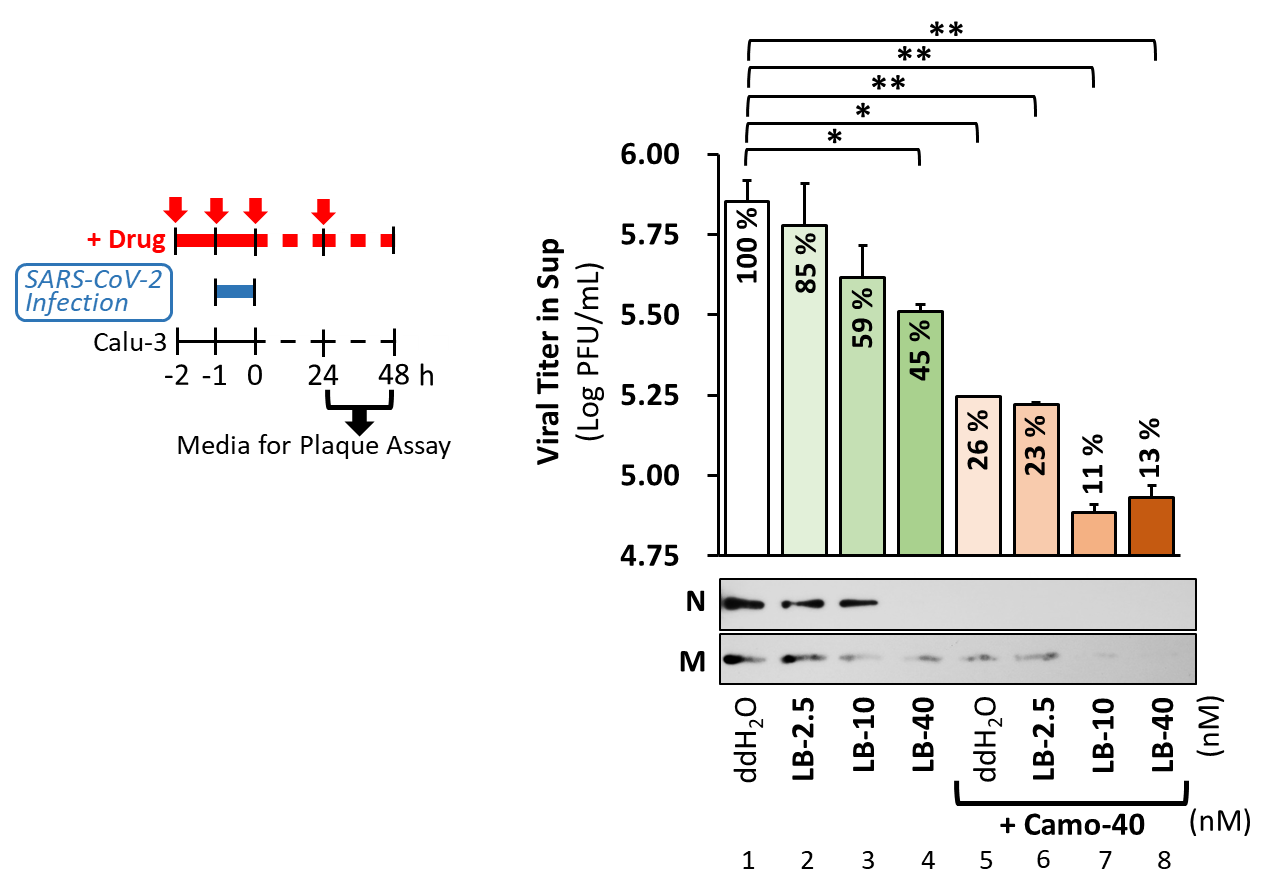
**

**Supplementary Figure 10. Combined Treatment of a PP2A Inhibitor and Camostat Displays Additive Anti-SARS-CoV-2 Effects.** The experimental workflow for determining the antiviral effect of LB-100 (LB) in combination with camostat (Camo) against SARS-CoV-2 in infected Calu-3 cells is shown (left diagram). Culture supernatants collected at 24–48 h post-infection were analyzed by plaque assay to determine viral load. Viral titers from two independent experiments are presented as log values (mean ± SD) of plaque-forming units (PFU) per mL, along with relative percentages compared with the ddH₂O control group (set to 100%) (right bar graph). The corresponding supernatants were also analyzed by western blotting to assess N and M protein levels in secreted virions; representative data from two independent experiments are shown (right panels). *p < 0.05; **p < 0.01.

**Supplementary Figure 11**

**
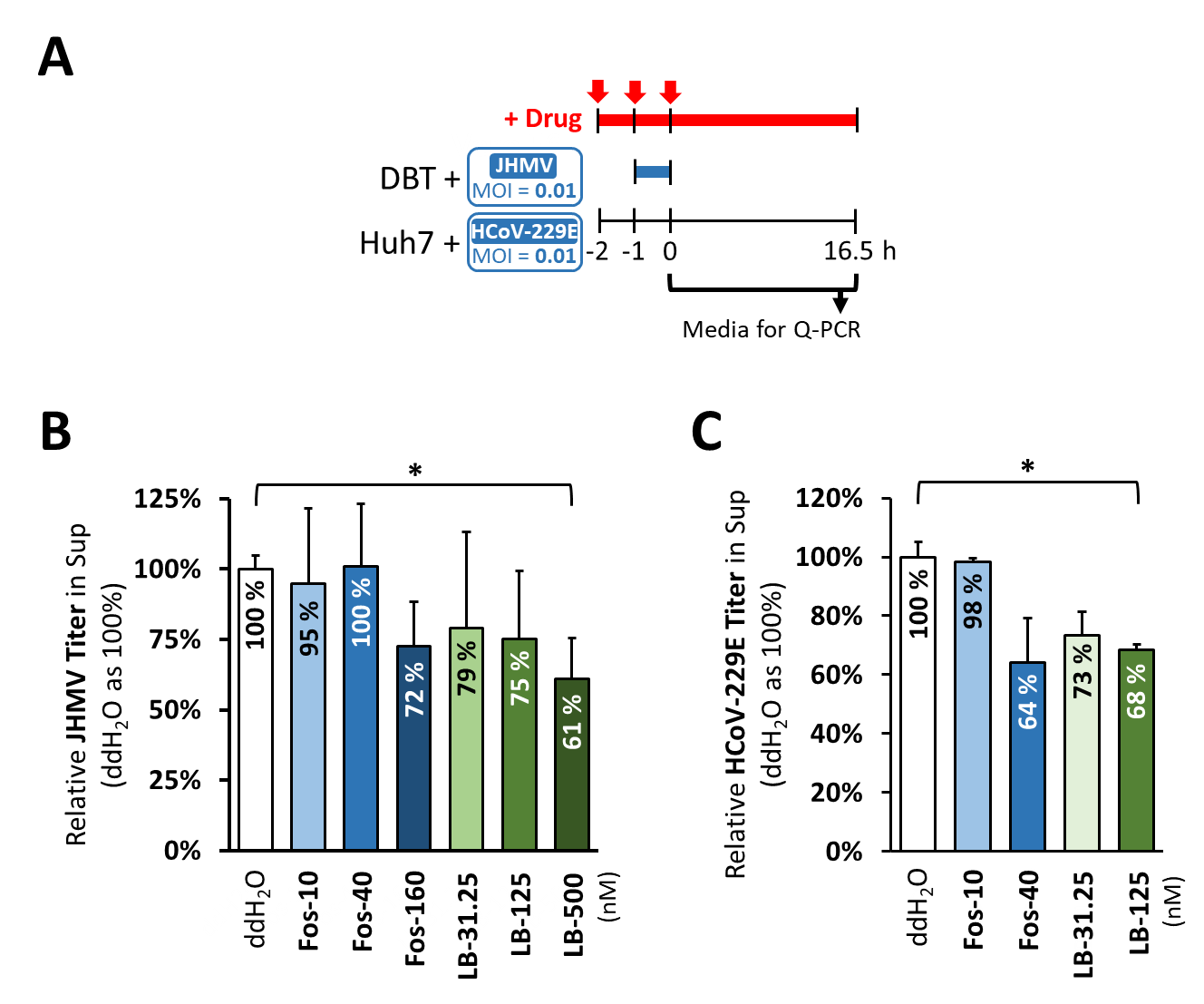
**

**Supplementary Figure 11. PP2A Inhibitors Display Antiviral Effects against JHMV and HCoV-229E.** **(A)** The experimental workflow for evaluating the antiviral effects of fostriecin (Fos) and LB-100 (LB) against JHMV and HCoV-229E is schematically illustrated. **(B**, **C)** Culture supernatants collected at 16.5 h post-infection were analyzed by RT–qPCR to determine viral load. Data from two independent experiments are presented as relative percentages, with the ddH₂O control group set to 100%. *p < 0.05.

**Supplementary Table 1: List of Applied Primer Sets.**

| **Primer Name** | **Primer Sequence** |
| --- | --- |
| N-S188A-F | 5’-CTCTTCTCGTTCCTCAGCACGTAGTCGCAACAG-3’ |
| N-S188A-R | 5’-CTGTTGCGACTACGTGCTGAGGAACGAGAAGAG-3’ |
| N-S206A-F | 5’-CAGCAGTAGGGGAACTGCTCCTGCTAGAATGGC-3’ |
| N-S206A-R | 5’-GCCATTCTAGCAGGAGCAGTTCCCCTACTGCTG-3’ |
| N-S188D-F | 5’- GCCTCTTCTCGTTCCTCAGATCGTAGTCGCAACAGTTCAAG-3' |
| N-S188D-R | 5’- CTTGAACTGTTGCGACTACGATCTGAGGAACGAGAAGAGGC-3' |
| N-S206D-F | 5'- GCAGCAGTAGGGGAACTGATCCTGCTAGAATGGCTGG-3' |
| N-S206D-R | 5'- CCAGCCATTCTAGCAGGATCAGTTCCCCTACTGCTGC -3' |
| E-Sarbeco-F | 5'- ACAGGTACGTTAATAGTTAATAGCGT -3' |
| E-Sarbeco-R | 5'- ATATTGCAGCAGTACGCACACA -3' |
| 20914-F | 5'- AGACAGTGGTTGCCTACGGG -3' |
| 21223-R | 5'- ATGCGAAGTGTCCCATGAGC -3' |
| JHMV-F | 5'- ATGTCTTTTGTTCCTGGGCAA -3' |
| JHMV-R | 5'- CCGGAATTGGGTTGAGTAGT -3' |
| 229E-1ab-F | 5'- GCATATGGCTGTTCTACTATTGCGC -3' |
| 229E-1ab-R | 5'- CCAACCACCCATGAAGCATAAAAGG -3' |
| PBGD-1070F | 5’- CATGAAGATGGCCCTGAGGAT -3’ |
| PBGD-1266R | 5’- CTGGTTTGTGGGGCACAGATGCC -3’ |
| CoV-N-1F | 5'- AAGCTGGACTTCCCTATGGTGC -3' |
| CoV-N-1R | 5'- CCTTGGGTTTGTTCTGGACCACG -3' |
| PBGD-F | 5'- GGTGACCAGCACACTTTGGG -3' |
| PBGD-R | 5'- AGCCGGGTGTTGAGGTTTCC -3' |

**Supplementary Table 2: List of Information for Key Resources Used**

| *Reagent or Resource* | *Source* | *Identifier* |
| --- | --- | --- |
| ***Antibodies*** | | |
| Rabbit anti-SCoV-N | Wu et al. ^6^ | N/A |
| Rabbit anti-phospho-SCoV-N (S177) | Wu et al. ^6^ | N/A |
| Rabbit anti-SARS-CoV-2 Spike | Genetex | GTX632604 |
| Rabbit anti-SARS-CoV-2 E | Genetex | GTX136046 |
| Rabbit anti-SARS-CoV-2 Nsp3 | Genetex | GTX135589 |
| Rabbit anti-FASN | Genetex | GTX109833 |
| Rabbit anti-PPP2R1A | Genetex | GTX102206 |
| Rabbit anti-PPP1C | Genetex | GTX105295 |
| Rabbit anti-PK | Genetex | GTX107546 |
| Rabbit anti-SARS-CoV-2 M | Novus Biologicals | NBP3-05698 |
| Mouse anti-PPP2C | Sigma-Aldrich | SAB1411750 |
| Mouse anti-β-Actin | Sigma-Aldrich | A5441 |
| Rabbit anti-phospho-Akt (T308) | Cell Signaling | # 4056 |
| Rabbit anti-pan Akt | Cell Signaling | # 4691 |
| Rabbit anti-phospho-GSK-3β (S9) | Cell Signaling | # 9336 |
| Rabbit anti-GSK-3β | Cell Signaling | # 9315 |
| Rabbit anti-HA (F-7) | Santa Cruz | sc-7392 |
| Mouse anti-RTN3 (F-6) | Santa Cruz | sc-374599 |
| Mouse anti-ERGIC-53 (F-3) | Santa Cruz | sc-398777 |
| Mouse anti-β-Catenin | BD Biosciences | # 610154 |
| Mouse anti-active-β-Catenin (8E7) | Merck Millipore | # 05-665 |
| ***Virus Strain*** | | |
| hCoV-19/Taiwan/NTU03/2020 | Laboratory of Sui-Yuan Chang | GISAID (Accession  ID:EPI_ISL_413592) |
| hCoV-19/Taiwan/NTU17/2020 | Laboratory of Sui-Yuan Chang | GISAID (Accession  ID:EPI_ISL_422419) |
| hCoV-19/Taiwan/NTU37/2020 | Laboratory of Sui-Yuan Chang | GISAID (Accession  ID:EPI_ISL_740547) |
| hCoV-19/Taiwan/NTU52/2021 | Laboratory of Sui-Yuan Chang | GISAID (Accession  ID:EPI_ISL_1041958) |
| hCoV-19/Taiwan/NTU54/2021 | Laboratory of Sui-Yuan Chang | GISAID (Accession  ID:EPI_ISL_1039160) |
| hCoV-19/Taiwan/NTU61/2021 | Laboratory of Sui-Yuan Chang | GISAID (Accession  ID:EPI_ISL_1667474) |
| hCoV-19/Taiwan/NTU62/2021 | Laboratory of Sui-Yuan Chang | GISAID (Accession  ID:EPI_ISL_1667475) |
| ***Continued*** | | |
| *Reagent or Resource* | *Source* | *Identifier* |
| hCoV-19/Taiwan/NTU96/2021 | Laboratory of Sui-Yuan Chang | N/A |
| hCoV-19/Taiwan/NTU110/2021 | Laboratory of Sui-Yuan Chang | GISAID (Accession  ID:EPI_ISL_11362243) |
| hCoV-19/Taiwan/NTU119/2021 | Laboratory of Sui-Yuan Chang | N/A |
| hCoV-19/Taiwan/NTU121/2021 | Laboratory of Sui-Yuan Chang | N/A |
| hCoV-19/Taiwan/NTU128/2021 | Laboratory of Sui-Yuan Chang | GISAID: (Accession ID:EPI_ISL_11050301) |
| hCoV-19/Taiwan/NTU142/2022 | Laboratory of Sui-Yuan Chang | GISAID: (Accession ID:EPI_ISL_13105945) |
| hCoV-19/Taiwan/NTU293/2023 | Laboratory of Sui-Yuan Chang | GISAID: (Accession ID:EPI_ISL_18278880) |
| JHMV | Laboratory of Michael M. C. Lai | N/A |
| HCoV-229E | Laboratory of Sui-Yuan Chang | N/A |
| ***Chemicals and Reagents*** | | |
| 6-bromoindirubin-30-oxime | MedChemExpress | HY-10580 |
| LY2090314 | MedChemExpress | HY-16294 |
| Kenpaullone | MedChemExpress | HY-12302 |
| LB-100 | MedChemExpress | HY-18597 |
| Tautomycin | MedChemExpress | HY-12728 |
| Okadaic acid | Tocris Bioscience | # 1136 |
| Fostriecin sodium salt | Tocris Bioscience | # 1840 |
| Lipofectamine 2000 | Thermo Fisher Scientific | # 11668019 |
| Alkaline Phosphatase | Thermo Fisher Scientific | EF0651 |
| Bis-Tris NuPAGE (10%, 1.0-mm) | Thermo Fisher Scientific | NP0301BOX |
| RIPA Lysis Buffer (10x) | Merck Millipore | # 20-188 |
| Phosphatase Inhibitor Cocktail II | Merck Millipore | # 524625 |
| Micrococcal Nuclease | New England Biolabs | M0247 |
| Lambda DNA HindIII Digest | New England Biolabs | N3012 |
| ***Continued*** | | |
| *Reagent or Resource* | *Source* | *Identifier* |
| Protease Inhibitor Cocktail | Roche Diagnostics | # 04693132001 |
| Passive Lysis Buffer (5x) | Promega | E1941 |
| ***Critical Commercial Assays*** | | |
| QuikChange II XL Site-Directed Mutagenesis Kit | Agilent | # 200521 |
| Dual-Luciferase Reporter Assay System | Promega | E1910 |
| Subcellular Protein Fractionation Kit | Thermo Fisher Scientific | # 78840 |
| SuperScript™ III First-Strand Synthesis System | Thermo Fisher Scientific | # 18080051 |
| BCA Protein Assay Kit | Thermo Fisher Scientific | # 23225 |
| NucleoSpin RNA, Mini kit for RNA purification | Macherey-Nagel | # 740955.50 |
| Nylon membrane, positively charged | Roche Diagnostics | # 11209299001 |
| DIG Wash and Block Buffer set | Roche Diagnostics | # 11585762001 |
| Anti-Digoxigenin-AP Fab Fragments | Roche Diagnostics | # 11093274910 |
| CDP-Star, Ready-to-Use | Roche Diagnostics | # 12041677001 |
| PCR DIG probe synthesis kit | Roche Diagnostics | # 11636090910 |
| ***Experimental Models: Cell Lines*** | | |
| Vero E6 | ATCC | CRL-1586 |
| 293T | ATCC | CRL-3216 |
| 293T/hACE2 stable cell | Laboratory of Mi-Hao Tao | N/A |
| DBT | Wu et al. ^6^ | N/A |
| Huh7 | Yang et al. ^50^ | N/A |
| ***Oligonucleotides*** | | |
| N-S188A-F | This paper | N/A |
| N-S188A-R | This paper | N/A |
| N-S206A-F | This paper | N/A |
| N-S206A-R | This paper | N/A |
| N-S188D-F | This paper | N/A |
| N-S188D-R | This paper | N/A |
| N-S206D-F | This paper | N/A |
| N-S206D-R | This paper | N/A |
| E-Sarbeco-F | Corman et al. ^51^ | N/A |
| E-Sarbeco-R | Corman et al. ^51^ | N/A |
| ***Continued*** | | |
| *Reagent or Resource* | *Source* | *Identifier* |
| 20914-F | This paper | N/A |
| 21223-R | This paper | N/A |
| JHMV-F | This paper | N/A |
| JHMV-R | This paper | N/A |
| 229E-1ab-F | This paper | N/A |
| 229E-1ab-R | This paper | N/A |
| CoV-N-1F | Cheng et al. ^52^ | N/A |
| CoV-N-1R | Cheng et al. ^52^ | N/A |
| PBGD-F | Cheng et al. ^52^ | N/A |
| PBGD-R | Cheng et al. ^52^ | N/A |
| PBGD-1070F | This paper | N/A |
| PBGD-1266R | This paper | N/A |
| ***Recombinant DNA*** | | |
| pCMV-Tag2B-SCoV N | Wu et al. ^6^ | N/A |
| pcDNA3.1(+)-SARS-CoV-2 N | This paper | N/A |
| pcDNA3.1(+)-SARS-CoV-2 N-S188D/S206D | This paper | N/A |
| pcDNA3.1(+)-SARS-CoV-2 N-S188A/S206A | This paper | N/A |
| pLVX-EF1alpha-SARS-CoV-2-M | Laboratory of Che Ma | N/A |
| pLVX-EF1alpha-SARS-CoV-2-E | Laboratory of Che Ma | N/A |
| pcDNA-3xHA-CoV2-M-WT | This paper | N/A |
| pcDNA-3xHA-CoV2-M-∆195-222 | This paper | N/A |
| pcDNA-3xHA-CoV2-M-∆159-222 | This paper | N/A |
| pcDNA-3xHA-CoV2-M-∆119-222 | This paper | N/A |
| pcDNA-3xHA-CoV2-M-∆119-158 | This paper | N/A |
| CoV2-M-IRES-E | Addgene | # 177938 |
| pUC57-2019-nCoV-S-D614G (humanized) | Cheng et al. ^52^ | N/A |
| pUC57-2019-nCoV-S-D614G (humanized, w/o HA-tag) | This paper | N/A |
| Luc-T20 | Addgene | # 177941 |
| pLKO.1-shLuc | National RNAi Core Facility | TRCN0000072246 |
| pLKO.1-PPP2C | National RNAi Core Facility | TRCN0000002483 |
| pLKO.1-PPP1C | National RNAi Core Facility | TRCN0000002455 |
| pLKO.1-PPP2R1A-clone 1 | National RNAi Core Facility | TRCN0000231600 |
| ***Continued*** |  |  |
| *Reagent or Resource* | *Source* | *Identifier* |
| pLKO.1-PPP2R1A-clone 2 | National RNAi Core Facility | TRCN0000231508 |
| pLKO.1-PPP2R1A-clone 3 | National RNAi Core Facility | TRCN0000231509 |
| pCMV-dR8.91 | National RNAi Core Facility | N/A |
| pMD.G | National RNAi Core Facility | N/A |
| ***Protein Sequence*** | | |
| SARS-CoV-2 M | Uniprot | P0DTC5 |
| SARS-CoV-2 N | Uniprot | P0DTC9 |
| PP2A catalytic subunit | Uniprot | #3KQ51 |
| PP2A scaffold subunit | Uniprot | C9J9C1 |
| SARS-CoV-1 M | Uniprot | P59596 |
| SARS-CoV-1 N | Uniprot | P59595 |
| JHMV M | Uniprot | P08549 |
| JHMV N | Uniprot | P03417 |
| HCoV-229E M | Uniprot | A0A1L7B920 |
| HCoV-229E N | Uniprot | P15130 |
| Mouse PP2A catalytic subunit | Uniprot | P63330 |
| Mouse PP2A scaffold subunit | Uniprot | Q76MZ3 |
| ***Software and Algorithm*** | | |
| ImageJ | Java source | https://imagej.net/ij/download.html |
| ChimearaX (version 1.8) | UCSF | https://www.rbvi.ucsf.edu/chimerax |

**Supplementary Table 3: Summary of Applied Dosages and Reported Cytotoxicity of Inhibitors**


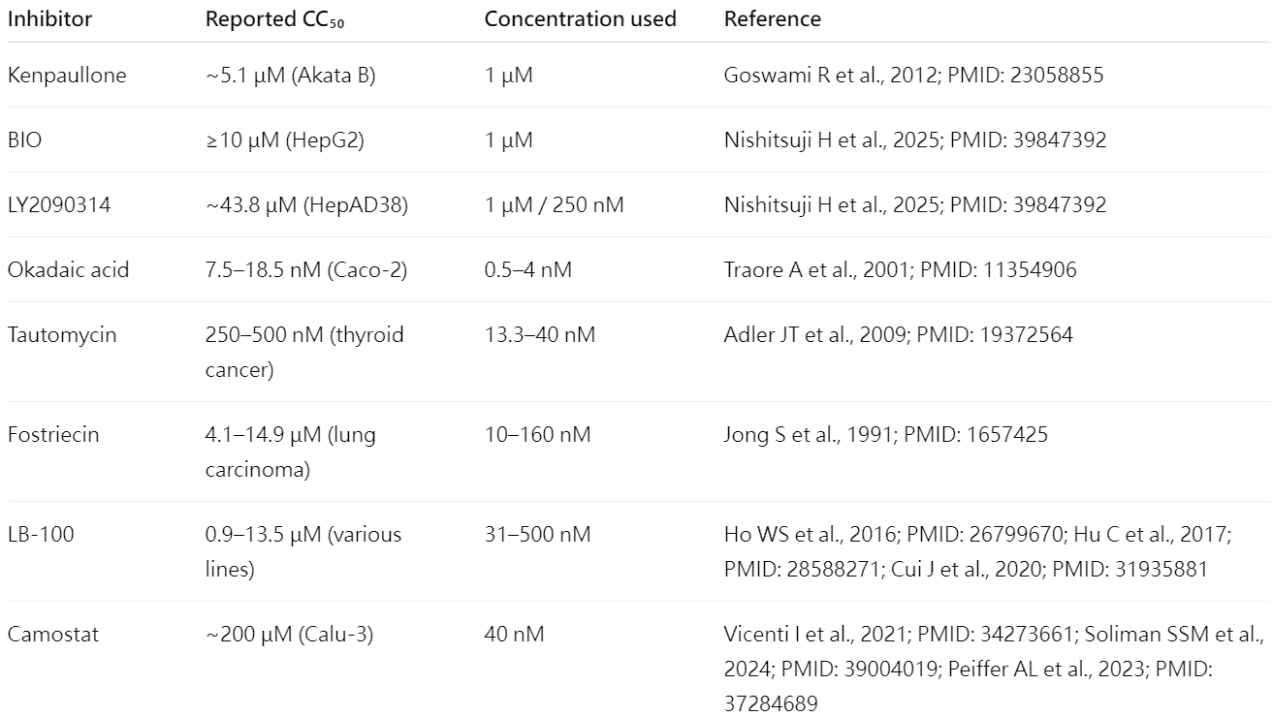

Supplement: Supplementary file 1 [file 12929_2026_1255_MOESM1_ESM.docx]
